# Pharmacologic Potential of Statins in Cancer Prevention: Colo-Rectal Cancer Risk in Dyslipidemic Patients from a Korean Nationwide Cohort

**DOI:** 10.3390/ph18081236

**Published:** 2025-08-21

**Authors:** Ho Suk Kang, Joo-Hee Kim, Heejin Kim, Joong Seob Lee, Hyo Geun Choi, Dae Myoung Yoo, Kyeong Min Han, Nan Young Kim, Kyueng-Whan Min, Mi Jung Kwon

**Affiliations:** 1 Division of Gastroenterology, Department of Internal Medicine, Hallym University Sacred Heart Hospital, Hallym University College of Medicine, Anyang 14068, Republic of Korea; hskang76@hallym.or.kr; 2 Division of Pulmonary, Allergy, and Critical Care Medicine, Department of Internal Medicine, Hallym University Sacred Heart Hospital, Hallym University College of Medicine, Anyang 14068, Republic of Korea; luxjhee@hallym.or.kr; 3 Department of Otorhinolaryngology-Head & Neck Surgery, Hallym University Sacred Heart Hospital, Anyang 14068, Republic of Korea; mir5020@hallym.or.kr (H.K.); apniosio@naver.com (J.S.L.); 4 Suseo Seoul E.N.T. Clinic, 10, Bamgogae-ro 1-gil, Gangnam-gu, Seoul 06349, Republic of Korea; mdanalytics@naver.com; 5 Hallym Data Science Laboratory, Hallym University College of Medicine, Anyang 14068, Republic of Korea; ydm@hallym.ac.kr (D.M.Y.); km.han@hallym.ac.kr (K.M.H.); 6 Hallym Institute of Translational Genomics and Bioinformatics, Hallym University Medical Center, Anyang 14068, Republic of Korea; honeyny@hallym.or.kr; 7 Department of Pathology, Uijeongbu Eulji Medical Center, Eulji University School of Medicine, 712, Dongil-ro, Uijeongbu 11759, Republic of Korea; kyueng@gmail.com; 8 Department of Pathology, Hallym University Sacred Heart Hospital, Hallym University College of Medicine, Anyang 14068, Republic of Korea

**Keywords:** colorectal cancer, incidence, mortality risk, lipophilic statins, hydrophobic statins, nationwide case–control study

## Abstract

**Background/Objectives**: Colorectal cancer (CRC) is a growing public health concern in South Korea, with incidence rising alongside dyslipidemia. Statins, widely prescribed for lipid control, have been proposed to reduce CRC risk, but evidence remains inconsistent, particularly in Asian populations. **Methods**: Using Korean National Health Insurance Service data (2002–2019), we conducted a nested case–control study of 9920 CRC patients and 39,680 matched controls. To reduce confounding, we applied a matching process with a propensity score and overlap weighting based on demographic and clinical variables. Statin use within two years before CRC diagnosis was categorized by type (lipophilic vs. hydrophilic) and duration. Lifestyle data such as smoking and diet were not available. **Results**: Short-term statin use was associated with a 17% reduced CRC risk, particularly in younger, metabolically healthier Korean males. Lipophilic statins were consistently associated with lower CRC risk and mortality. However, hydrophilic statins showed mixed results: while short-term use lowered CRC risk, long-term use was linked to increased all-cause mortality. These associations varied by patient subgroup. **Conclusion**: Among Korean adults, short-term statin use—especially lipophilic agents—was associated with favorable CRC outcomes. However, the observational design, the absence of lifestyle data, and increased mortality linked to long-term hydrophilic statin use limit causal interpretation. Further research using clinically enriched or prospective datasets is warranted to validate these findings and guide personalized preventive strategies.

## 1. Introduction

Colorectal cancer (CRC) is a significant global health burden. In the United States, it ranks as the third most commonly diagnosed cancer and the second leading cause of cancer-related mortality, with over 150,000 new cases projected in 2024 [1]. In South Korea, CRC has rapidly become the second most common cancer and the third leading cause of cancer-related death [2]. The age-standardized CRC incidence in Korea more than doubled from 1999 to 2014, driven by demographic aging, urbanization, and dietary Westernization [2,3,4]. Despite early improvements in five-year survival—attributable to national screening programs and treatment advances—this progress has plateaued since the mid-2010s [5]. The continued burden of CRC in Korea highlights the importance of effective prevention strategies, particularly among older adults with multiple comorbidities.

Parallel to the rise in CRC, the prevalence of dyslipidemia has markedly increased in Korea, growing by over 55% between 2007 and 2020 and now affecting nearly half of adults aged ≥ 30 years [6]. The increase has been most notable among older adults and those in rural areas, where access to preventive care may be limited [6,7]. Despite the availability of statins and national guidelines, the awareness and treatment rates for dyslipidemia in Korea remain low, especially in high-risk groups such as the elderly [6]. Given the high prevalence of both CRC and dyslipidemia [6], exploring the potential role of statins in CRC prevention is of particular relevance to Korean public health [5].

Statins, the most widely prescribed lipid-lowering agents, inhibit 3-hydroxy-3-methylglutaryl-coenzyme A reductase and have been hypothesized to exert anticancer effects beyond cardiovascular protection [8]. Mechanistically, statins may interfere with cancer-related signaling through the mevalonate pathway, and experimental studies suggest they can reduce tumor proliferation, angiogenesis, and inflammation [9,10,11,12,13]. However, while these biological pathways offer plausible explanations for a protective effect, most evidence remains preclinical. Translating these findings into clinical relevance requires population-based studies that assess outcomes in real-world settings, particularly in diverse populations [14,15,16,17].

Epidemiologic studies examining the association between statin use and CRC risk have yielded mixed results [18,19,20,21,22,23,24,25,26]. Several observational studies and meta-analyses have reported a reduced CRC incidence and improved survival among statin users [18,19,20,21], especially with lipophilic statins or in specific subgroups [22,23,24,25,26,27]. However, the evidence is less consistent in Asian populations [14,15,16,17]. For example, two Taiwanese studies and one Japanese study did not observe a significant association between statin use and CRC risk [15,16,17]. These null findings may be attributed to limitations such as the inclusion of only female participants [15], the use of special patient populations (e.g., glaucoma patients) [17], or insufficient adjustment for key confounders such as socioeconomic status and comorbidities [15,16,17]. Moreover, heterogeneous definitions of statin exposure and lack of stratification by statin type or duration complicate interpretation [15,16,17,18,19,20,21,22,23,24,25,26]. One Korean study using national data did report a reduced incidence of gastrointestinal cancers associated with lipid-lowering therapies, but it did not isolate CRC outcomes, nor did it stratify by statin subtype [14].

Pharmacologic differences between statin types may contribute to these inconsistencies [16,17,27,28,29,30,31]. Statins can be classified as lipophilic (e.g., simvastatin, atorvastatin) or hydrophilic (e.g., rosuvastatin, pravastatin) based on solubility and tissue permeability profiles [32]. Lipophilic statins penetrate extrahepatic tissues more readily and may exert broader systemic effects, whereas hydrophilic statins are more liver-selective and may have limited distribution to colorectal tissue [28,29,30,32]. Several studies have suggested differences in anticancer efficacy based on solubility, but the evidence remains inconclusive and largely derived from Western cohorts [16,17,27,31]. Additionally, statin treatment duration may influence outcomes, with potential for either beneficial or paradoxical effects depending on exposure length and patient characteristics.

To address this gap, we conducted a population-based, nested case–control study using Korean national health data to investigate whether demographic and clinical characteristics modify the association between statin use and CRC incidence and mortality, while accounting for statin type (lipophilic vs. hydrophilic) and duration of use. Through this study, we aim to clarify the potential role of statins in CRC prevention and survivorship, with the broader goal of informing future risk-adapted strategies in population-level cancer control.

## 2. Results

### 2.1. Baseline Characteristics

After performing 1:4 propensity score matching, 9920 CRC patients and 39,680 matched controls were included in the final analysis. The subsequent application of overlap weighting resulted in well-balanced demographic and clinical characteristics between the two groups (standardized difference = 0), including mean age distribution, sex ratio, income classes, region of residence, Charlson Comorbidity Index (CCI), diabetes, and dyslipidemia histories (Table 1).

### 2.2. Statin Exposure Among Study Participants

The distribution of statin exposure among patients with CRC and matched controls was summarized (Table S1). After applying propensity score overlap weighting, statin prescription patterns were well balanced between the two groups, with standardized differences ≤ 0.07 across all categories, indicating good covariate comparability.

Overall, 24.2% of CRC patients and 22.7% of controls had been prescribed any statins. Short-term use (<180 days) was observed in 6.92% of CRC patients and 5.99% of controls, intermediate-term use (180–545 days) in 6.74% vs. 6.48%, and long-term use (≥545 days) in 10.53% vs. 10.21%, respectively—demonstrating comparable distributions between groups.

When stratified by statin type, 19.36% of CRC patients and 18.23% of controls used lipophilic statins, while 7.16% of CRC patients and 6.85% of controls used hydrophilic statins. Exposure durations were similarly distributed across both groups.

These findings indicate high post-matching comparability in statin exposure between CRC and control groups, supporting the robustness of subsequent outcome analyses.

### 2.3. CRC Incidence and Statin Use

We investigated the odds ratios (ORs) of incident CRC based on the duration and type of statin use (Table 2). The use of any statin correlated with a significant reduction in CRC likelihood across all durations: <180 days (OR = 0.83, 95% confidence interval (CI): 0.77–0.90, *p* < 0.001), 180–545 days (OR = 0.92, 95% CI: 0.85–0.99, *p* = 0.033), and ≥545 days (OR = 0.93, 95% CI: 0.87–0.99, *p* = 0.021). Similarly, both lipophilic and hydrophilic statins were associated with significantly reduced CRC odds when used for <180 days (lipophilic: OR = 0.88, 95% CI: 0.81–0.95; hydrophilic: OR = 0.86, 95% CI: 0.76–0.97).

### 2.4. Subgroup Analyses: Statin Use and CRC Incidence

Intermediate-term statin use (180–545 days) was associated with a lower likelihood of CRC, particularly among individuals with higher incomes.

**Figure 1:**
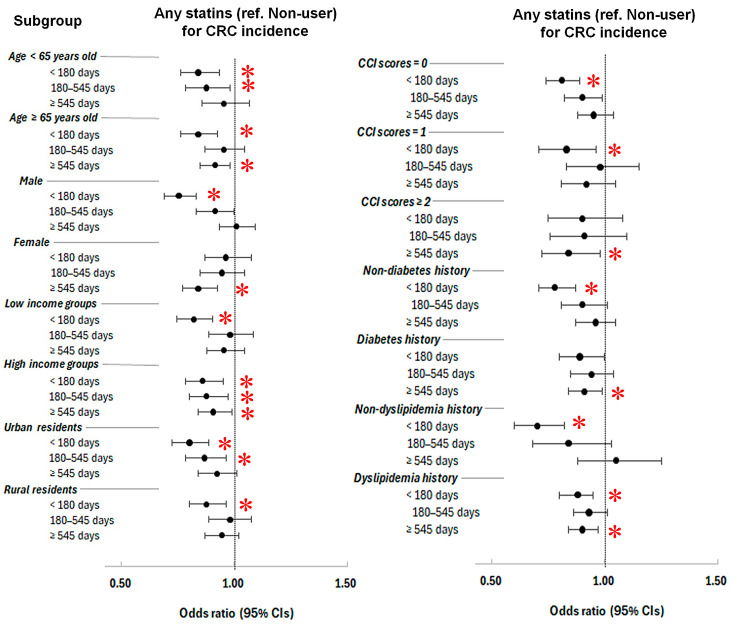
Forest plot of overlap-weighted odds ratios (95% confidence intervals (CIs) presented with bar and dot for CRC incidence by duration of any statin use across subgroups. Short-term statin use (<180 days) was consistently associated with reduced CRC risk across most populations. Long-term use (≥545 days) was linked to = lower likelihood of CRC occurrence in higher-risk groups, including older adults, females, high-income individuals, and those with greater comorbidity (CCI ≥ 2). * Significance at *p* <0.05.

Notably, long-term any statin use (≥545 days) conferred a reduced likelihood of CRC occurrence in higher-risk subgroups such as older adults, females, individuals with high incomes, and those with multiple comorbidities (CCI ≥ 2), diabetes, or dyslipidemia (Figure 1 and Table S2).

In lipophilic statins, short-term use (<180 days) correlated with a lessened likelihood of CRC occurrence, being evident among males, younger individuals (<65 years), those with high incomes, urban residents, people with no comorbidities (CCI = 0), and those with no diabetes or no dyslipidemia history (Table S3). Intermediate-term use (180–545 days) was linked with a lessened likelihood of incident CRC, being evident among individuals less than age 65 years, urban residents, those with no comorbidities (CCI = 0), and those with no diabetes. The long-term use of lipophilic statins was also related to a reduced likelihood of CRC development, affecting females, those on high incomes, and those with the presence of dyslipidemia history.

In contrast, the short-term use of hydrophilic statins (<180 days) reduced CRC probability in older adults, males, and individuals with low-income status, urban residents, no comorbidity, high comorbidity burden, or lower income levels. The long-term use of hydrophilic statins was also associated with a reduced likelihood of CRC occurrence, including in females and high comorbidity burden (CCI ≥ 2). Interestingly, the intermediate-term use of hydrophilic statins was conversely linked with increased likelihood of incident CRC, including low-income status, low comorbidity burden (CCI = 1), and individuals without diabetes (Table S4).

### 2.5. All-Cause Mortality in CRC Patients

We evaluated the association between statin use and mortality among patients with CRC, considering both the duration of use and statin type (lipophilic vs. hydrophilic) (Table 3). Short-term use of any statin (<180 days) was significantly associated with reduced mortality likelihood (OR = 0.78, 95% CI: 0.66–0.92, *p* = 0.003). In contrast, long-term any statin use (≥545 days) was associated with an increased likelihood of mortality (OR = 1.22, 95% CI: 1.06–1.41, *p* = 0.007).

Notable variations in mortality likelihood were observed depending on the type of statin and the duration of treatment. When analyzed by statin type, short-term and intermediate-term lipophilic statin use was associated with decreased mortality: <180 days (OR = 0.69, 95% CI: 0.58–0.82, *p* < 0.001) and 180–545 days (OR = 0.80, 95% CI: 0.67–0.95, *p* = 0.011).

In contrast, hydrophilic statins consistently showed a significant association with increased mortality likelihood across all durations: <180 days (OR = 1.41, 95% CI: 1.08–1.84, *p* = 0.011), 180–545 days (OR = 1.72, 95% CI: 1.28–2.32, *p* < 0.001), and ≥545 days (OR = 1.81, 95% CI: 1.38–2.38, *p* < 0.001).

### 2.6. Subgroup Analyses: Statin Use and CRC Mortality

Subgroup analyses revealed that the short-term use of any statin (<180 days) was consistently associated with reduced mortality in multiple populations, including those aged < 65 years (OR = 0.73, 95% CI = 0.57–0.93, *p* = 0.012), males (OR = 0.77, 95% CI = 0.62–0.95, *p* = 0.016), individuals with low-income group (OR = 0.75, 95% CI = 0.60–0.95, *p* = 0.017), rural residents (OR = 0.68, 95% CI = 0.54–0.85, *p* < 0.001), those with a low CCI score of 0 or 1, and participants without diabetes (OR = 0.77, 95% CI = 0.62–0.97, *p* = 0.025), regardless of lipidemia history.

**Figure 2:**
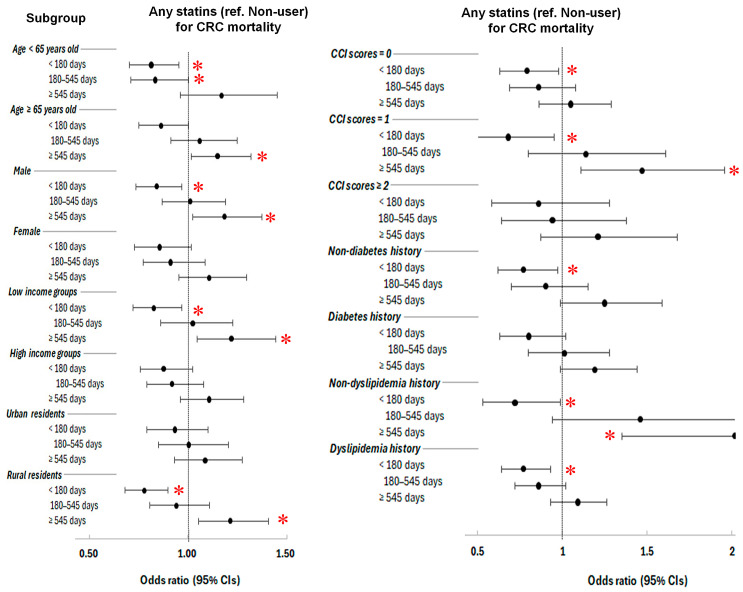
Forest plots of overlap-weighted odds ratios (95% confidence intervals (CIs) presented with bar and dot for colorectal cancer (CRC) mortality according to duration of any statin use across patient subgroups. Short-term statin use (<180 days) was associated with reduced CRC mortality likelihood in younger adults, males, low-income and rural populations, those with low CCI, and non-diabetic individuals. Long-term use (≥545 days) was linked to increased mortality likelihood in older adults, males, low-income and rural residents, those with CCI = 1, and individuals without dyslipidemia. * Significance at *p* < 0.05.

In contrast, the long-term use of any statins (≥545 days) correlated with increased mortality likelihood in some high-risk subgroups, such as individuals aged ≥ 65 years (OR = 1.21, 95% CI = 1.02–1.45, *p* = 0.032), males (OR = 1.26, 95% CI = 1.03–1.53, *p* = 0.023), low-income groups (OR = 1.31, 95% CI = 1.60–1.63, *p* = 0.013), rural residents (OR = 1.30, 95% CI = 1.07–1.58, *p* = 0.008), those with a CCI of 1 (OR = 1.47, 95% CI = 1.11–1.96, *p* = 0.007), and those with no dyslipidemia history (OR = 2.02, 95% CI = 1.35–3.02, *p* < 0.001) (Figure 2 and Table S5).

For lipophilic statins (Table S6), short-term use for <180 days significantly reduced mortality likelihood in several groups, independent of age, sex, income, residence area, diabetes or dyslipidemia history. This included individuals with a low CCI score of 0 or 1. The intermediate-term use of lipophilic statins (180–545 days) also conferred reduced mortality likelihood in certain subgroup, such as individuals with younger age (<65 years), females, high-income group, rural residents, a CCI of 0, individuals with no diabetes, and individuals with dyslipidemia history.

Conversely, the short-term use of hydrophilic statins (<180 days) was associated with the increased likelihood of CRC mortality, in subgroups of older age (age ≥ 65 years), females, low incomes, urban residents, CCI = 0, the presence of diabetes history, or with dyslipidemia history. The intermediate-term use of hydrophilic statins (180–545 days) was linked with increased likelihood of CRC mortality, independent age, income status, histories of diabetes or dyslipidemia history, affecting females, rural residents, and CCI = 0.

Notably, the long-term use (≥545 days) of hydrophilic statins was associated with a significantly increased risk of all-cause mortality (OR = 1.81, 95% CI = 1.38–2.38, *p* < 0.001). This increased mortality likelihood of CRC was found independent age, sex, residence areas, or histories of diabetes or dyslipidemia history. This included patients with a high-income status, a CCI = 0, or a high comorbidity burden (CCI ≥ 2) (Table S7).

## 3. Discussion

In this large Korean nested case–control study (*n* = 49,600), prior statin use was associated with reduced colorectal cancer (CRC) incidence, with variable effects on all-cause mortality depending on statin type, duration, and patient characteristics. Short-term statin use (<180 days), especially lipophilic agents, consistently reduced both CRC incidence and mortality, with greater benefits among younger, healthier males. Intermediate- and long-term use also lowered CRC risk, particularly among older adults with metabolic disorders. However, long-term use—especially of hydrophilic statins—was unexpectedly associated with increased all-cause mortality in high-risk groups, including normolipidemic, comorbid elderly individuals.

Overall, any statin use was associated with a 17% lower odds of incident CRC compared to non-use (OR = 0.83), with even short-term use (<180 days) showing a statistically significant but modest protective effect. This association persisted across several subgroups, including younger individuals (<65 years), males, rural or low-income residents, and those with fewer comorbidities. Notably, short-term lipophilic statin use was linked to reduced CRC risk particularly among metabolically healthier individuals without diabetes, dyslipidemia, or multiple comorbid conditions. While the observed effect size was relatively small, such findings may still have meaningful public health implications given the high prevalence of statin use and the global burden of CRC. Moreover, preclinical studies have demonstrated that statins can induce early tumor-suppressive processes—including the inhibition of cell proliferation, angiogenesis, and inflammatory signaling—as early as within days of exposure [9,11,13]. For instance, simvastatin has been shown to impair spheroid formation in ovarian cancer stem cells and trigger oxidative stress-induced apoptosis in p53-deficient colon cancer cells within 1–3 days by disrupting nucleotide biosynthesis [13,33]. Similarly, fluvastatin has demonstrated up to 70% inhibition of in vitro tumor cell growth and reduced metastasis over 12 days in animal models [9]. These findings may support the biological plausibility of early anticancer effects, particularly among metabolically stable individuals [31], although the clinical relevance of such short-term exposure warrants further investigation in prospective and mechanistic studies.

Intermediate- and long-term any statin use were also associated with reduced CRC risk in our study. These findings echo earlier reports from the UK and Spain, which noted decreased CRC incidence with prolonged statin use (≥1 year) [22,34]. A UK study reported a 36% reduction in CRC risk with statin use exceeding one year [22], and a Spanish study showed a similar benefit for up to three years of use (OR = 0.79) [34], corresponding roughly to the intermediate and long-term periods considered in our study. However, both studies lacked stratified analyses by statin type or detailed subgroup investigations. In our analysis, significant subgroup differences emerged between intermediate and long-term statin users. While intermediate-term users resembled short-term users—showing benefits primarily in younger, healthier populations—long-term users showed a stronger protective effect in elderly, high-comorbidity patients, particularly women with metabolic disorders. In women, declining estrogen levels after menopause contribute to rising cholesterol levels with age, increasing cardiovascular risk [35]. Prior studies have also noted that statins can lower both cholesterol levels and cancer risk, particularly in elderly patients with metabolic diseases like diabetes [19,36]. These findings may indicate that sustained use may be more helpful in controlling CRC development among high-risk populations [37].

When examining mortality outcomes, we found that short-term statin use was associated with significantly lower all-cause mortality among CRC patients (OR = 0.78), with the strongest benefits observed in younger males, rural residents, individuals with fewer comorbidities, and those without diabetes. Lipophilic statins, in particular, demonstrated consistent survival benefits, as both short- and intermediate-term use were linked to reduced mortality—supporting their potential systemic anticancer activity beyond lipid regulation. These findings are generally consistent with prior meta-analyses, including a 2019 pooled analysis of over 130,000 CRC patients and a 2021 Chinese meta-analysis, both of which reported reductions in cancer-specific and all-cause mortality with statin use [38,39]. However, our study also revealed an unexpected association between long-term statin use (≥545 days) and increased all-cause mortality (OR = 1.22), with the most pronounced effect observed for long-term hydrophilic statin use (OR = 1.81). These findings were especially evident among older adults, males, individuals with higher comorbidity burdens, and those without a history of dyslipidemia. This apparent contradiction with the prior literature—which generally reported protective or null effects—should be interpreted with caution. Importantly, previous studies did not stratify outcomes by statin type, duration, or clinical subgroups, limiting comparability. In contrast, our large, stratified Korean cohort enabled the identification of nuanced temporal and pharmacologic patterns that may have been masked in aggregated analyses. Nonetheless, despite applying rigorous statistical methods—including propensity score matching and overlap weighting—residual confounding remains a concern. Long-term hydrophilic statins may be more frequently prescribed to frail or older patients with higher cardiovascular risk, and unmeasured factors such as medication adherence, treatment intent, or clinical frailty could have influenced the observed associations. Additionally, cause-specific mortality data were not available in the KNHIS-NSC, limiting our ability to determine whether deaths were cancer-related or due to other causes.

Several biological mechanisms may also help contextualize our findings. Prolonged statin exposure may not confer indefinite chemopreventive benefits and, in certain settings, could exert unintended pro-tumor effects. For example, long-term statin use—particularly at low doses—has been shown to paradoxically promote tumor angiogenesis through upregulation of vascular endothelial growth factor and endothelial nitric oxide synthase pathways [40,41,42]. In normocholesterolemic conditions, simvastatin has been observed to activate Akt signaling and promote angiogenesis in animal models [43]. Furthermore, in patients without hyperlipidemia, chronic cholesterol suppression may impair membrane integrity, immune surveillance, and tissue repair mechanisms, potentially increasing vulnerability to cancer progression [44]. Moreover, transcriptomic analyses have demonstrated that statins broadly modulate gene expression in tumor cells, influencing pathways related to apoptosis, cell cycle, metabolism, and immune regulation in a highly context-dependent manner—varying by statin type, dosage, tissue environment, and host metabolic state [45]. Taken together, while lipophilic statins appear to offer short- to intermediate-term benefits, long-term use—particularly of hydrophilic agents—may be associated with increased mortality in certain high-risk subgroups. These results underscore the need for individualized clinical decision-making and highlight the importance of further prospective and mechanistic studies to clarify the long-term impact of statins on CRC survival.

In our study, both lipophilic and hydrophilic statins were associated with a reduced risk of CRC occurrence when used short-term. This protective effect may be attributed to multiple antitumor mechanisms beyond the classical mevalonate pathway, including the induction of apoptosis, the suppression of PI3K/Akt signaling, the enhancement of the p53/p21 pathway, the upregulation of p27 via EZH2 inhibition, and the modulation of Ras signaling [46,47,48]. Statins have also been shown to influence tumor behavior through alternative, cholesterol-independent pathways, such as the regulation of inflammatory cytokines, immune cell activity, oxidative stress responses, or global gene expression modulation [49,50,51].

Interestingly, the magnitude of CRC risk reduction observed with lipophilic statins was not consistently greater than that of hydrophilic statins, which contrasts with several earlier studies reporting superior outcomes with lipophilic agents [48,52]. These differences may be partially explained by pharmacokinetic and pharmacodynamic properties. Lipophilic statins passively diffuse across lipid membranes, enabling broader systemic distribution and greater access to extrahepatic tissues such as the colorectal epithelium [13]. They are primarily metabolized by membrane-bound cytochrome P450 enzymes, which may render them more effective in individuals with intact metabolic function [32]. In contrast, hydrophilic statins depend on active hepatic transporters for uptake and are predominantly excreted unchanged, limiting their distribution to peripheral tissues [30,32]. These pharmacokinetic differences suggest that statin type may influence anticancer efficacy depending on tumor location and host metabolic status [28,29,30]. Lipophilic statins have demonstrated greater effectiveness against nonhepatic solid tumors, such as breast and ovarian cancers [28,29], and may confer similar—though variably reported—benefits in CRC [16,17,27,31]. These pleiotropic anticancer effects may be particularly pronounced in metabolically stable individuals [28], which could help explain the enhanced protective effect observed in our study.

Conversely, hydrophilic statins (e.g., pravastatin, rosuvastatin) demonstrated a more complex pattern: while short-term use was associated with reduced CRC incidence, intermediate- and long-term use paradoxically showed an association with increased all-cause mortality. This apparent contradiction may reflect multiple factors. First, hydrophilic statins exhibit limited tissue distribution due to their reliance on active hepatic transporters, potentially restricting their impact on colorectal tumor microenvironments or systemic oncogenic pathways [13,30]. Second, confounding by indication may have influenced these findings, as hydrophilic statins are often prescribed to older individuals or those with higher comorbidity burdens—groups inherently at an elevated risk of mortality regardless of statin exposure [13,30,32,53]. Despite our efforts to adjust for these covariates, residual confounding may persist. Moreover, molecular evidence supports this pharmacologic distinction: hydrophilic statins show minimal modulation of gene expression profiles related to antiproliferative or pro-apoptotic activity [45], which may further limit their long-term chemopreventive efficacy. Thus, the increased mortality observed with prolonged hydrophilic statin use may be more reflective of underlying patient characteristics and limited tissue-level effects rather than a direct adverse consequence of the drug itself [13,30].

The strength of our study lies in its comprehensive evaluation of the association between prior statin use and CRC incidence and mortality in a large, stratified Korean population, incorporating detailed subgroup analyses by statin type and duration. Methodologically, the use of the KNHIS–NSC, a nationally representative dataset derived from a 2% stratified random sample of the Korean population [54], enhances the external validity of our findings. This database provides longitudinal data on diagnoses, prescriptions, sociodemographic characteristics, and mortality, making it well-suited for pharmacoepidemiologic research [54,55]. Furthermore, we applied rigorous analytic techniques—including 1:4 exact matching based on age, sex, income level, and region of residence—and utilized the index date to ensure covariate balance between 9920 CRC patients and 39,680 matched controls. After this matching step, we estimated propensity scores using the full set of baseline covariates and applied overlap weighting to further adjust for residual confounding and achieve covariate balance. The achieved covariate balance (absolute standardized differences ≤ 0.2) approximates conditions of a randomized trial and reduces confounding [56,57]. The high comparability in statin exposure (24.2% in CRC patients vs. 22.7% in controls) and demographic characteristics between groups may further help support the internal validity of our findings.

In contrast, many previous studies have reported inconsistent associations between statin use and CRC risk, often influenced by factors such as age, comorbidity burden, or specific clinical conditions like inflammatory bowel disease [17,28,58]. However, these studies frequently lacked rigorous adjustment for socioeconomic and clinical confounders [15,16,17,28,59], thereby limiting the interpretability of their results. Moreover, earlier research frequently employed undefined statin exposure windows [15,22,30,34,59,60] or included only CRC patients as cases with unmatched controls [22,24,30], increasing the risk of selection bias and confounding by indication.

Recent large cohort studies and updated meta-analyses, including Asian populations, further underscore these methodological challenges and mixed findings [15,16,17,31,39,61]. For example, two Taiwanese studies found no significant association between statin use and CRC incidence, but lacked adjustment for treatment duration and metabolic status—factors shown to significantly influence outcomes [15,16]. Similarly, a Japanese cohort study conducted in patients with glaucoma failed to identify a protective effect, though its limited generalizability and imbalanced cohort design reduce confidence in its conclusions [31]. In contrast, a recent comprehensive meta-analysis, based on 52 observational studies, confirmed a statistically significant reduction in CRC risk among statin users (relative risk = 0.88), with stronger effects observed for long-term use and lipophilic statins [39]. These results may reinforce our findings regarding the possible chemopreventive potential of statins, particularly the importance of statin type and treatment duration. Furthermore, a large U.S.-based prospective study (ARIC) found no significant increase in CRC incidence or mortality among statin users [61], supporting our conclusion that statins may not elevate CRC risk—and may even reduce mortality when used on a short- to mid-term basis, especially with lipophilic agents. Notably, however, ARIC’s cohort was predominantly White and African American, limiting its applicability to Asian populations [61]. Our analysis extends these findings by providing detailed stratification based on statin type, duration of use, and metabolic profile in a large Korean cohort, offering important insights into the evolving understanding of statins in cancer prevention—particularly within an Asian context where both CRC incidence and statin use are high.

One important limitation of this study is its reliance on the Korean National Health Insurance Service–National Sample Cohort (KNHIS-NSC), an administrative claims database primarily designed for healthcare management rather than clinical research. While this cohort provides a large, nationally representative sample with long-term follow-up, it lacks detailed clinical information such as cancer staging, genetic profiles, tumor characteristics, and concurrent treatments (e.g., chemotherapy). Lifestyle factors—including smoking, alcohol consumption, physical activity, body mass index, and diet—were also unavailable in the administrative data. Although we adjusted for available clinical and socioeconomic variables, residual confounding may persist due to unmeasured risk factors such as family history, medication adherence, and health behaviors. In particular, our definition of statin exposure was based solely on prescription claims without verification of actual medication use, raising the potential for exposure misclassification that could attenuate or bias effect estimates. Additionally, although Korea’s relatively homogeneous healthcare access and population structure may enhance internal validity, the single-country design may limit generalizability to ethnically diverse or non-Asian populations. The protective associations observed may not extrapolate to populations with differing metabolic profiles, statin prescription patterns, or CRC screening practices.

Furthermore, the retrospective observational design may inherently limit causal inference. Although we employed rigorous analytic methods—including 1:4 propensity score matching and overlap weighting—to minimize confounding and selection bias, residual confounding and temporal ambiguity cannot be fully excluded. This limitation is particularly relevant for long-term statin users, who may have had greater baseline comorbidity, frailty, or polypharmacy, potentially confounding the observed association with increased all-cause mortality [13,32,33]. As such, these mortality findings should be interpreted with caution, as they may reflect underlying patient health status rather than a direct adverse effect of statin use. In addition, our mortality outcome was restricted to all-cause death, as cause-specific mortality data were not available in the KNHIS–NSC database. This limitation precluded us from determining whether the increased mortality observed among certain statin users was attributable to cancer progression, cardiovascular events, or other unrelated causes.

Lastly, the multiple substratified analyses—although clinically meaningful—may have reduced statistical power in some subgroups, increasing the risk of false-positive or -negative findings. Despite these limitations, the use of a large-scale, population-based cohort and advanced statistical adjustments supports the robustness of our main findings. Nonetheless, future studies using clinically enriched datasets (e.g., NHIS-NHID, KCPS-II, KoGES) with linked mortality and adherence data—or ideally, prospective cohort studies and randomized controlled trials—are warranted to confirm these associations and clarify their causal implications.

## 4. Materials and Methods

### 4.1. Data Source and Study Population

This retrospective, nested case–control study was approved by the Institutional Ethics Committee of Hallym University Sacred Heart Hospital (IRB No. 2022-10-008), with consent waived due to the use of de-identified data. Data were drawn from the KNHIS-NSC, a representative sample of approximately 2.2% of the Korean population tracked from 2002 to 2019 [54,55]. CRC cases were identified from 2005 to 2019 based on the simultaneous the presence of both ICD-10 diagnostic codes (C18–C20) and national cancer-specific billing codes (V193 or V194). The ICD-10 codes represent malignant neoplasms of the colon (C18), rectosigmoid junction (C19), and rectum (C20), while the V-codes are only assigned to patients with officially confirmed cancer diagnoses who are registered in the Korean national insurance system to receive financial support for critical illnesses. This dual-coding strategy is commonly employed in Korean administrative database studies to improve diagnostic specificity and enhance case ascertainment by reducing the likelihood of including patients with only suspected or provisional cancer diagnoses [62].

**Figure 3:**
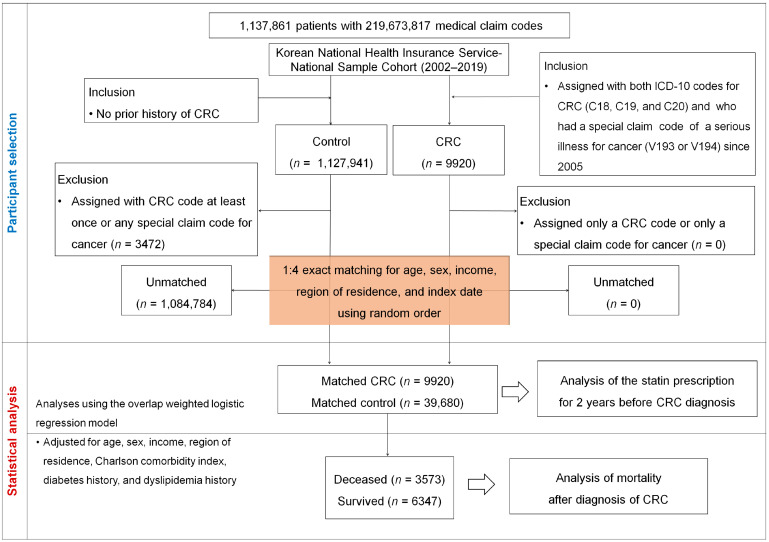
The flow diagram of participant selection and study design. This study utilized data from the Korean National Health Insurance Service–National Sample Cohort (2002–2019), comprising 1,137,861 individuals with over 219 million medical claim records. Individuals with colorectal cancer (CRC) were identified based on concurrent ICD-10 codes (C18, C19, and C20) and a special claim code for serious cancer illness (V193 or V194). Control participants were selected from individuals without a history of CRC or cancer-related claim codes. After exclusions and a 1:4 exact matching by age, sex, income, region of residence, and index date, a total of 9920 CRC cases and 39,680 matched controls were included. Statin use was assessed for two years prior to CRC diagnosis, and participants were further classified by survival status for mortality analysis.

Controls were individuals without CRC or a serious cancer code during the same period. After excluding 3472 potential misclassified individuals, 1,124,469 remained eligible. We first performed 1:4 exact matching based on age, sex, income level, and region of residence. The index date for each CRC patient—defined as the date of all assigned diagnosis codes and the V-codes—was assigned to a randomly selected matched control. As the index date of the control participants followed the index date of their matched patients with CRC, each patient with CRC and matched control member had the same index date, yielding 39,680 matched controls. After this matching step, we estimated propensity scores using the full set of baseline covariates and applied overlap weighting to further adjust for residual confounding and achieve covariate balance. Ultimately, 9920 CRC cases and 39,680 controls were included for the analysis of statin exposure within two years prior to diagnosis and subsequent survival outcomes (Figure 3). The primary outcome was CRC incidence based on statin exposure (type and duration). The secondary outcome was all-cause mortality among CRC patients.

### 4.2. Definition of Statin Exposure

Statin exposure was assessed within the two-year window prior to the index date, referencing prior studies demonstrating early cardiovascular and potential antitumor benefits within this period [63]. Exposure duration was categorized as short-term (<180 days), intermediate-term (180–545 days), or long-term (≥545 days) exposure. These cutoffs were selected based on pharmacodynamic and epidemiological evidence. Statins typically begin exerting lipid-lowering effects within several weeks of initiation, whereas their anti-inflammatory and potential anticancer effects are thought to require longer exposure—often exceeding one year [17,25,36,62,64]. The <180-day threshold has been widely adopted in prior cancer pharmacoepidemiologic studies as an operational definition of short-term use, reflecting minimal systemic exposure [65]. Conversely, ≥545 days (approximately 1.5 years) has been suggested as the minimum duration necessary for sustained modulation of tumor-related pathways and tissue-level chemopreventive effects [17,25,39,62,64]. We included an intermediate exposure category (180–545 days) to assess potential dose-response patterns or transitional effects. These duration-based thresholds were further informed by a recent large-scale Korean study on statin use and gastrointestinal cancer risk [62], providing consistency with prior literature and enabling meaningful cross-study comparisons.

The statins included were atorvastatin, simvastatin, lovastatin, pravastatin, fluvastatin, and rosuvastatin. Statins were classified by lipophilicity—lipophilic (atorvastatin, simvastatin, lovastatin, and fluvastatin) and hydrophilic (pravastatin and rosuvastatin)—consistent with prior classifications [30,32,62]. Although fluvastatin is less lipophilic than other agents in this group, it demonstrates passive membrane permeability and a broad tissue distribution more similar to other lipophilic statins than to hydrophilic ones [30,32,62], justifying its grouping.

### 4.3. Covariates and Comorbidity Adjustment

Participants were stratified into 18 age groups with 5-year intervals and further categorized into 5 income classes ranging from class 1 (lowest income) to class 5 (highest income). Following our previous study, residential areas were classified as either urban or rural.

To assess participants’ overall disease burden, we used the CCI, a widely validated tool that quantifies comorbidity based on 17 predefined conditions, with scores ranging from 0 to 29 [65]. These conditions include myocardial infarction, heart failure, peripheral vascular disease, cerebrovascular disease, dementia, chronic pulmonary disease, connective tissue disorders, peptic ulcer, liver disease, diabetes (with and without complications), paraplegia, renal disease, cancer, metastatic cancer, severe liver disease, and HIV/AIDS. Diabetes (ICD-10 codes E10–E14) and dyslipidemia (E78) were identified based on diagnostic codes recorded at least twice. The CCI was calculated without CRC. The CCI score was treated as a continuous variable and used to approximate overall health status and frailty—factors known to influence cancer risk and outcomes, especially in older or hospitalized populations [65]. Given the potential for comorbidities to confound associations between statin use and CRC outcomes, we adjusted for CCI scores and individual comorbidities as covariates in our analyses. To further reduce residual confounding and enhance comparability between groups, we employed overlap weighting alongside multivariable conditional logistic regression models.

### 4.4. Statistical Analyses

We calculated propensity scores using multivariable logistic regression based on covariates: age, sex, income level, region of residence, and comorbidities included in the CCI. Using these scores, we performed 1:4 nearest-neighbor matching between CRC cases and controls. To further reduce confounding, overlap weighting was applied—weighting cases by (1–propensity score) and controls by the propensity score—ensuring all weights fell between 0 and 1 [56,66].

Covariate balance was assessed using absolute standardized differences, with ≤0.20 indicating sufficient matching quality [56]. We then used multivariable logistic regression models with overlap weighting to estimate crude and adjusted ORs with 95% CIs for CRC incidence and all-cause mortality, based on statin type and duration. Subgroup analyses were also conducted across covariate strata.

To test the robustness of our findings, we conducted sensitivity analyses by excluding (1) participants with any prior non-CRC cancer, and (2) those with high comorbidity burden (CCI ≥ 6). Results remained consistent with the primary analyses.

All statistical tests were two-tailed, and *p*-values < 0.05 were considered indicative of statistical significance. Analyses were conducted using SAS version 9.4 (SAS Institute Inc., Cary, NC, USA).

## 5. Conclusions

In summary, short-term statin use—particularly with lipophilic agents—was associated with reduced CRC incidence and all-cause mortality, especially among metabolically healthier individuals in this large, population-based Korean cohort. Conversely, long-term statin use, particularly hydrophilic statins, was associated with increased all-cause mortality in certain high-risk subgroups. While these findings may suggest a potential role for statins in CRC risk modulation, they must be interpreted cautiously given the observational nature of the study, the absence of cancer staging or adherence data, and potential residual confounding. Our results underscore the importance of further validation through prospective cohort studies and randomized controlled trials before clinical recommendations or personalized statin strategies can be implemented.

## Figures and Tables

**Table 1:** General characteristics of participants.

Characteristic	Before PS Overlap-Weighting Adjustment			After PS Overlap-Weighting Adjustment		
	CRC Group	Controls	SD	CRC Group	Controls	SD
Age (years) (n, %)			0.00			0.00
0–4	1 (0.01)	4 (0.01)		1 (0.01)	1 (0.01)	
5–9	N/A	N/A		N/A	N/A	
10–14	3 (0.03)	12 (0.03)		2 (0.03)	2 (0.03)	
15–19	1 (0.01)	4 (0.01)		1 (0.01)	1 (0.01)	
20–24	8 (0.08)	32 (0.08)		6 (0.08)	6 (0.08)	
25–29	26 (0.26)	104 (0.26)		21 (0.26)	21 (0.26)	
30–34	94 (0.95)	376 (0.95)		75 (0.95)	75 (0.95)	
35–39	180 (1.81)	720 (1.81)		144 (1.81)	144 (1.81)	
40–44	359 (3.62)	1436 (3.62)		287 (3.62)	287 (3.62)	
45–49	578 (5.83)	2312 (5.83)		462 (5.83)	462 (5.83)	
50–54	968 (9.76)	3872 (9.76)		773 (9.75)	773 (9.75)	
55–59	1242 (12.52)	4968 (12.52)		992 (12.52)	992 (12.52)	
60–64	1393 (14.04)	5572 (14.04)		1113 (14.04)	1113 (14.04)	
65–69	1488 (15.00)	5952 (15.00)		1189 (15.00)	1189 (15.00)	
70–74	1471 (14.83)	5884 (14.83)		1175 (14.83)	1175 (14.83)	
75–79	1060 (10.69)	4240 (10.69)		848 (10.69)	848 (10.69)	
80–84	672 (6.77)	2688 (6.77)		538 (6.78)	538 (6.78)	
85+	376 (3.79)	1504 (3.79)		301 (3.79)	301 (3.79)	
Sex (n, %)			0.00			0.00
Male	5933 (59.81)	23,732 (59.81)		4741 (59.81)	4741 (59.81)	
Female	3987 (40.19)	15,948 (40.19)		3186 (40.19)	3186 (40.19)	
Income (n, %)			0.00			0.00
1 (lowest)	1990 (20.06)	7960 (20.06)		1591 (20.07)	1591 (20.07)	
2	1253 (12.63)	5012 (12.63)		1001 (12.63)	1001 (12.63)	
3	1562 (15.75)	6248 (15.75)		1247 (15.73)	1247 (15.73)	
4	2059 (20.76)	8236 (20.76)		1646 (20.76)	1646 (20.76)	
5 (highest)	3056 (30.81)	12,224 (30.81)		2442 (30.81)	2442 (30.81)	
Region of residence (n, %)			0.00			0.00
Urban	4447 (44.83)	17,788 (44.83)		3554 (44.83)	3554 (44.83)	
Rural	5473 (55.17)	21,892 (55.17)		4373 (55.17)	4373 (55.17)	
CCI (mean, Sd)	0.58 (0.98)	0.53 (0.98)	0.05	0.57 (0.86)	0.57 (0.46)	0.00
Diabetes history (n, %)	3554 (35.83)	13,201 (33.27)	0.05	2799 (35.31)	2799 (35.31)	0.00
Dyslipidemia history (n, %)	4599 (46.36)	18,863 (47.54)	0.02	3693 (46.59)	3693 (46.59)	0.00

Abbreviations: PS, propensity score; CRC, colorectal cancer; SD, standardized difference; N/A, not applicable; CCI, Charlson Comorbidity Index; Sd, standard deviation.

**Table 2:** Crude and propensity score overlap-weighted odds ratios for colorectal cancer according to duration and type of statin use.

Characteristics	N of CRC Group	N of Controls	ORs for CRC(95% CI)	
	(exposure/total, %)	(exposure/total, %)	Overlap-weighted model†	*p*
Any statin types				
Non-user	7521/9920 (75.82)	30,667/39,680 (77.29)	1	
<180 days	686/9920 (6.92)	2382/39,680 (6.00)	0.83 (0.77–0.90)	<0.001 *
180–545 days	668/9920 (6.73)	2581/39,680 (6.50)	0.92 (0.85–0.99)	0.033 *
≥ 545 days	1045/9920 (10.53)	4050/39,680 (10.21)	0.93 (0.87–0.99)	0.021 *
Lipophilic statin type				
Non-user	8000/9920 (80.65)	32,449/39,680 (81.78)	1	
<180 days	610/9920 (6.15)	2206/39,680 (5.56)	0.88 (0.81–0.95)	0.001 *
180–545 days	581/9920 (5.86)	2224/39,680 (5.60)	0.93 (0.86–1.00)	0.063
≥545 days	729/9920 (7.35)	2801/39,680 (7.06)	0.93 (0.87–1.00)	0.063
Hydrophilic statin type				
Non-user	9210/9920 (92.84)	36,950/39,680 (93.12)	1	
<180 days	246/9920 (2.48)	857/39,680 (2.16)	0.86 (0.76–0.97)	0.012 *
180–545 days	218/9920 (2.20)	914/39,680 (2.30)	1.04 (0.92–1.17)	0.558
≥545 days	246/9920 (2.48)	959/39,680 (2.42)	0.97 (0.86–1.09)	0.575

Abbreviations: CRC, colorectal cancer; OR, odds ratios; 95% CI, 95% confidence interval. * Significance at *p* <0.05. †Adjusted for age, sex, income, region of residence, diabetes history, CCI, diabetes history, and dyslipidemia history.

**Table 3:** Crude and overlap propensity score–weighted odds ratios (ORs) for all-cause mortality in colorectal cancer patients according to duration and type of statin use.

Characteristics	Patients Who Died	Patients Who Survived	ORs for Mortality (95% CI)	
	(exposure/total, %)	(exposure/total, %)	Overlap-weighted model ^†^	*p*
Any statin use				
Non-user	2766/3573 (77.41)	4755/6347 (74.92)	1	
<180 days	260/3573 (7.28)	426/6347 (6.71)	0.78 (0.66–0.92)	0.003 *
180–545 days	215/3573 (6.02)	453/6347 (7.14)	0.96 (0.81–1.14)	0.629
≥545 days	332/3573 (9.29)	713/6347 (11.23)	1.22 (1.06–1.41)	0.007 *
Lipophilic statin use				
Non-user	2886/3573 (80.77)	5114/6347 (80.57)	1	
<180 days	240/3573 (6.72)	370/6347 (5.83)	0.69 (0.58–0.82)	<0.001 *
180–545 days	201/3573 (5.63)	380/6347 (5.99)	0.80 (0.67–0.95)	0.011 *
≥545 days	246/3573 (6.88)	483/6347 (7.61)	1.01 (0.86–1.18)	0.942
Hydrophilic statin use				
Non-user	3384/3573 (94.71)	5826/6347 (91.79)	1	
<180 days	72/3573 (2.02)	174/6347 (2.74)	1.41 (1.08–1.84)	0.011 *
180–545 days	49/3573 (1.37)	169/6347 (2.66)	1.72 (1.28–2.32)	<0.001 *
≥545 days	68/3573 (1.90)	178/6,347 (2.80)	1.81 (1.38–2.38)	<0.001 *

Abbreviations: 95% CI, 95% confidence interval. * Significance at *p* < 0.05. † Adjusted for age, sex, income, region of residence, diabetes history, CCI, diabetes history, and dyslipidemia history.

## Data Availability

Restrictions apply to the availability of these data. Data were obtained from the Korean National Health Insurance Sharing Service (NHISS) and are available at https://nhiss.nhis.or.kr (accessed on 1 October 2024) with the permission of the NHIS.

## Supplementary Materials

The following supporting information can be downloaded at: https://www.mdpi.com/article/10.3390/ph18081236/s1, Table S1: Distribution of Statin Exposure Among Patients with Colorectal Cancer and Matched Controls Before and After Propensity Score Overlap Weighting; Table S2: Crude and overlap propensity score weighted odd ratios of dates of any statin prescription for colorectal cancer; Table S3: Crude and overlap propensity score weighted odd ratios of dates of lipophilic statin prescription for colorectal cancer; Table S4: Crude and overlap propensity score weighted odd ratios of dates of hydrophilic statin prescription for colorectal cancer; Table S5: Crude and overlap propensity score weighted odd ratios of dates of any statin prescription for mortality in colorectal cancer; Table S6: Crude and overlap propensity score weighted odd ratios of dates of lipophilic statin prescription for mortality in colorectal cancer; Table S7: Crude and overlap propensity score weighted odd ratios of dates of hydrophilic statin prescription for mortality in colorectal cancer.
