# Supplementary material for: Pharmacologic Potential of Statins in Cancer Prevention: Colo-Rectal Cancer Risk in Dyslipidemic Patients from a Korean Nationwide Cohort
Source: Pharmaceuticals (Basel). 2025 Aug 21;18(8):1236. doi: 10.3390/ph18081236 (PMC12389320; doi:10.3390/ph18081236)
Supplement: Supplementary file 1 [file pharmaceuticals-18-01236-s001.zip › pharmaceuticals-3777282-supplementary.pdf]

Supplementary table 1. Distribution of Statin Exposure Among Patients with Colorectal Cancer and Matched Controls Before and After Propensity Score Overlap Weighting.

Characteristic	Before PS overlap-weighting adjustment			After PS overlap-weighting adjustment		
	CRC group	Controls	SD	CRC group	Controls	SD
Any statin use (n, %)			0.04			0.07
Non-user	7521 (75.82)	30,667 (77.29)		6010 (75.81)	6129 (77.31)	
< 180 days	686 (6.92)	2382 (6.00)		549 (6.92)	475 (5.99)	
180–545 days	668 (6.73)	2581 (6.50)		534 (6.74)	514 (6.48)	
≥ 545 days	1045 (10.53)	4050 (10.21)		835 (10.53)	810 (10.21)	
Lipophilic statin use (n, %)			0.04			0.04
Non-user	8000 (80.65)	32,449 (81.78)		6393 (80.64)	6482 (81.77)	
< 180 days	610 (6.15)	2206 (5.56)		488 (6.15)	441 (5.56)	
180–545 days	581 (5.86)	2224 (5.60)		465 (5.86)	444 (5.60)	
≥ 545 days	729 (7.35)	2801 (7.06)		582 (7.35)	561 (7.07)	
Hydrophilic statin use (n, %)			0.00			0.00
Non-user	9210 (92.84)	36,950 (93.12)		7360 (92.84)	7384 (93.15)	
< 180 days	246 (2.48)	857 (2.16)		197 (2.48)	170 (2.15)	
180–545 days	218 (2.20)	914 (2.30)		174 (2.20)	182 (2.29)	
≥ 545 days	246 (2.48)	959 (2.42)		196 (2.48)	191 (2.41)	

Abbreviations: PS, propensity score; SD, standardized difference.

Supplementary table 2. Crude and overlap propensity score weighted odd ratios of dates of any statin prescription for colorectal cancer (CRC)

Characteristics	N of CRC	N of Control	ORs for CRC (95% CI)			
	(exposure/total, %)	(exposure/total, %)	Crude	p	Overlap weighted model †	p
Age < 65 years old (n=24,265)						
Non-user	3,987/4,853 (82.16)	16,311/19,412 (84.03)	1		1	
< 180 days	306/4,853 (6.31)	1,040/19,412 (5.36)	1.20 (1.05-1.37)	0.006*	0.83 (0.75-0.93)	0.001*
180–545 days	269/4,853 (5.54)	941/19,412 (4.85)	1.17 (1.02-1.35)	0.028*	0.87 (0.77-0.98)	0.018*
≥ 545 days	291/4,853 (6.00)	1,120/19,412 (5.77)	1.06 (0.93-1.21)	0.37	0.95 (0.85-1.07)	0.393
Age ≥ 65 years old (n=25,335)						
Non-user	3,534/5,067 (69.75)	14,356/20,268 (70.83)	1		1	
< 180 days	380/5,067 (7.50)	1,342/20,268 (6.62)	1.15 (1.02-1.30)	0.022*	0.83 (0.75-0.92)	<0.001*
180–545 days	399/5,067 (7.87)	1,640/20,268 (8.09)	0.99 (0.88-1.11)	0.842	0.95 (0.86-1.05)	0.330
≥ 545 days	754/5,067 (14.88)	2,930/20,268 (14.46)	1.05 (0.96-1.14)	0.324	0.91 (0.84-0.98)	0.012*
Male (n=29,665)						
Non-user	4,628/5,933 (78.00)	18,876/23,732 (79.54)	1		1	
< 180 days	407/5,933 (6.86)	1,249/23,732 (5.26)	1.33 (1.18-1.49)	<0.001*	0.74 (0.67-0.82)	<0.001*
180–545 days	352/5,933 (5.93)	1,320/23,732 (5.56)	1.09 (0.96-1.23)	0.177	0.91 (0.82-1.00)	0.062
≥ 545 days	546/5,933 (9.20)	2,287/23,732 (9.64)	0.97 (0.88-1.07)	0.597	1.01 (0.93-1.10)	0.762
Female (n=19,935)						
Non-user	2,893/3,987 (72.56)	11,791/15,948 (73.93)	1		1	
< 180 days	279/3,987 (7.00)	1,133/15,948 (7.10)	1.00 (0.88-1.15)	0.959	0.96 (0.86-1.08)	0.507
180–545 days	316/3,987 (7.93)	1,261/15,948 (7.91)	1.02 (0.90-1.16)	0.75	0.94 (0.84-1.05)	0.246
≥ 545 days	499/3,987 (12.52)	1,763/15,948 (11.05)	1.15 (1.04-1.28)	0.009*	0.83 (0.76-0.92)	<0.001*
Low income groups (n=24,025)						
Non-user	3,672/4,805 (76.42)	15,004/19,220 (78.06)	1		1	
< 180 days	346/4,805 (7.20)	1,140/19,220 (5.93)	1.24 (1.09-1.41)	<0.001*	0.81 (0.73-0.90)	<0.001*
180–545 days	314/4,805 (6.53)	1,246/19,220 (6.48)	1.03 (0.91-1.17)	0.656	0.98 (0.88-1.09)	0.736
≥ 545 days	473/4,805 (9.84)	1,830/19,220 (9.52)	1.06 (0.95-1.18)	0.319	0.95 (0.87-1.05)	0.331
High income groups (n=25,575)						
Non-user	3,849/5,115 (75.25)	15,663/20,460 (76.55)	1		1	
< 180 days	340/5,115 (6.65)	1,242/20,460 (6.07)	1.11 (0.98-1.26)	0.091	0.85 (0.77-0.95)	0.003*
180–545 days	354/5,115 (6.92)	1,335/20,460 (6.52)	1.08 (0.95-1.22)	0.223	0.87 (0.79-0.97)	0.009*
≥ 545 days	572/5,115 (11.18)	2,220/20,460 (10.85)	1.05 (0.95-1.16)	0.346	0.90 (0.83-0.99)	0.023*
Urban residents (n=22,235)						
Non-user	3,331/4,447 (74.9)	13,685/17,788 (76.93)	1		1	
< 180 days	316/4,447 (7.11)	1,059/17,788 (5.95)	1.23 (1.08-1.40)	0.002*	0.79 (0.71-0.88)	<0.001*
180–545 days	323/4,447 (7.26)	1,187/17,788 (6.67)	1.12 (0.98-1.27)	0.09	0.86 (0.77-0.96)	0.008*
≥ 545 days	477/4,447 (10.73)	1,857/17,788 (10.44)	1.06 (0.95-1.18)	0.326	0.92 (0.83-1.01)	0.068
Rural residents (n=27,365)						
Non-user	4,190/5,473 (76.56)	16,982/21,892 (77.57)	1		1	
< 180 days	370/5,473 (6.76)	1,323/21,892 (6.04)	1.13 (1.01-1.28)	0.041*	0.87 (0.79-0.96)	0.006*
180–545 days	345/5,473 (6.30)	1,394/21,892 (6.37)	1.00 (0.89-1.13)	0.961	0.98 (0.88-1.08)	0.651
≥ 545 days	568/5,473 (10.38)	2,193/21,892 (10.02)	1.05 (0.95-1.16)	0.333	0.94 (0.86-1.02)	0.14
CCI scores = 0 (n=33,441)						
Non-user	5,010/6,331 (79.13)	21,856/27,110 (80.62)	1		1	
< 180 days	384/6,331 (6.07)	1,404/27,110 (5.18)	1.19 (1.06-1.34)	0.003*	0.81 (0.74-0.89)	<0.001*
180–545 days	384/6,331 (6.07)	1,546/27,110 (5.70)	1.08 (0.96-1.22)	0.175	0.90 (0.82-0.99)	0.035*

≥ 545 days	553/6,331 (8.73)	2,304/27,110 (8.50)	1.05 (0.95-1.15)	0.356	0.95 (0.88-1.04)	0.264
CCI scores = 1 (n=10,037)						
Non-user	1,676/2,303 (72.77)	5,577/7,734 (72.11)	1		1	
< 180 days	184/2,303 (7.99)	558/7,734 (7.21)	1.10 (0.92-1.31)	0.299	0.83 (0.71-0.96)	0.016*
180–545 days	160/2,303 (6.95)	587/7,734 (7.59)	0.91 (0.76-1.09)	0.296	0.98 (0.83-1.15)	0.765
≥ 545 days	283/2,303 (12.29)	1,012/7,734 (13.09)	0.93 (0.81-1.07)	0.323	0.92 (0.81-1.05)	0.225
CCI scores ≥ 2 (n=6,122)						
Non-user	835/1,286 (64.93)	3,234/4,836 (66.87)	1		1	
< 180 days	118/1,286 (9.18)	420/4,836 (8.68)	1.09 (0.88-1.35)	0.447	0.90 (0.75-1.08)	0.256
180–545 days	124/1,286 (9.64)	448/4,836 (9.26)	1.07 (0.87-1.33)	0.522	0.91 (0.76-1.10)	0.335
≥ 545 days	209/1,286 (16.25)	734/4,836 (15.18)	1.10 (0.93-1.31)	0.263	0.84 (0.72-0.98)	0.025*
Non-diabetes history (n=32,845)						
Non-user	5,323/6,366 (83.62)	22,398/26,479 (84.59)	1		1	
< 180 days	370/6,366 (5.81)	1,274/26,479 (4.81)	1.22 (1.08-1.38)	0.001*	0.78 (0.71-0.87)	<0.001*
180–545 days	299/6,366 (4.7)	1,207/26,479 (4.56)	1.04 (0.92-1.19)	0.532	0.90 (0.81-1.01)	0.062
≥ 545 days	374/6,366 (5.87)	1,600/26,479 (6.04)	0.98 (0.88-1.11)	0.78	0.96 (0.87-1.05)	0.364
Diabetes history (n=16,755)						
Non-user	2,198/3,554 (61.85)	8,269/13,201 (62.64)	1		1	
< 180 days	316/3,554 (8.89)	1,108/13,201 (8.39)	1.07 (0.94-1.23)	0.302	0.89 (0.80-1.00)	0.052
180–545 days	369/3,554 (10.38)	1,374/13,201 (10.41)	1.01 (0.89-1.14)	0.871	0.94 (0.85-1.04)	0.239
≥ 545 days	671/3,554 (18.88)	2,450/13,201 (18.56)	1.03 (0.93-1.14)	0.548	0.91 (0.84-0.99)	0.036*
Non-dyslipidemia history (n=26,138)						
Non-user	4,965/5,321 (93.31)	19,707/20,817 (94.67)	1		1	
< 180 days	165/5,321 (3.1)	437/20,817 (2.1)	1.50 (1.25-1.80)	<0.001*	0.70 (0.60-0.82)	<0.001*
180–545 days	83/5,321 (1.56)	258/20,817 (1.24)	1.28 (1.00-1.64)	0.055	0.84 (0.68-1.03)	0.097
≥ 545 days	108/5,321 (2.03)	415/20,817 (1.99)	1.03 (0.83-1.28)	0.767	1.05 (0.88-1.25)	0.601
Dyslipidemia history (n=23,462)						
Non-user	2,556/4,599 (55.58)	10,960/18,863 (58.1)	1		1	
< 180 days	521/4,599 (11.33)	1,945/18,863 (10.31)	1.15 (1.03-1.28)	0.01*	0.88 (0.80-0.95)	0.002*
180–545 days	585/4,599 (12.72)	2,323/18,863 (12.32)	1.08 (0.98-1.19)	0.134	0.93 (0.86-1.01)	0.076
≥ 545 days	937/4,599 (20.37)	3,635/18,863 (19.27)	1.11 (1.02-1.20)	0.019*	0.90 (0.84-0.97)	0.005*

Abbreviations: CRC, colorectal cancer; OR, odds ratio; 95% CI, 95% confidence interval. * Significance at $p < 0.05$. †Adjusted for age, sex, income, region of residence, diabetes history, CCI, diabetes history, and dyslipidemia history.

Supplementary table 3. Crude and overlap propensity score weighted odd ratios of dates of lipophilic statin prescription for colorectal cancer (CRC).

Characteristics	N of CRC	N of Control	ORs for CRC (95% CI)			
	(exposure/total, %)	(exposure/total, %)	Crude	p	Overlap weighted model†	p
Age < 65 years old (n=24,265)						
Non-user	4,149/4,853 (85.49)	16,939/19,412 (87.26)	1		1	
< 180 days	273/4,853 (5.63)	921/19,412 (4.74)	1.21 (1.05-1.39)	0.007*	0.83 (0.74-0.94)	0.002*
180–545 days	232/4,853 (4.78)	794/19,412 (4.09)	1.19 (1.03-1.39)	0.021*	0.86 (0.75-0.97)	0.016*
≥ 545 days	199/4,853 (4.10)	758/19,412 (3.90)	1.07 (0.91-1.26)	0.395	0.94 (0.83-1.08)	0.39
Age ≥ 65 years old (n=25,335)						
Non-user	3,851/5,067 (76.00)	15,510/20,268 (76.52)	1		1	
< 180 days	337/5,067 (6.65)	1,285/20,268 (6.34)	1.06 (0.93-1.20)	0.391	0.91 (0.82-1.01)	0.087
180–545 days	349/5,067 (6.89)	1,430/20,268 (7.06)	0.98 (0.87-1.11)	0.783	0.97 (0.88-1.08)	0.616
≥ 545 days	530/5,067 (10.46)	2,043/20,268 (10.08)	1.04 (0.94-1.16)	0.399	0.92 (0.84-1.00)	0.063
Male (n=29,665)						
Non-user	4,891/5,933 (82.44)	19,920/23,732 (83.94)	1		1	
< 180 days	358/5,933 (6.03)	1,146/23,732 (4.83)	1.27 (1.13-1.44)	<0.001*	0.78 (0.70-0.86)	<0.001*
180–545 days	304/5,933 (5.12)	1,127/23,732 (4.75)	1.10 (0.96-1.25)	0.158	0.90 (0.81-1.01)	0.069
≥ 545 days	380/5,933 (6.4)	1,539/23,732 (6.48)	1.01 (0.89-1.13)	0.925	0.98 (0.89-1.08)	0.732
Female (n=19,935)						
Non-user	3,109/3,987 (77.98)	12,529/15,948 (78.56)	1		1	
< 180 days	252/3,987 (6.32)	1,060/15,948 (6.65)	0.96 (0.83-1.11)	0.557	1.03 (0.91-1.15)	0.66
180–545 days	277/3,987 (6.95)	1,097/15,948 (6.88)	1.02 (0.89-1.17)	0.804	0.96 (0.85-1.07)	0.457
≥ 545 days	349/3,987 (8.75)	1,262/15,948 (7.91)	1.11 (0.98-1.26)	0.089	0.88 (0.79-0.98)	0.023*
Low income groups (n=24,025)						
Non-user	3,902/4,805 (81.21)	15,795/19,220 (82.18)	1		1	
< 180 days	298/4,805 (6.20)	1,074/19,220 (5.59)	1.12 (0.98-1.28)	0.087	0.91 (0.81-1.01)	0.085
180–545 days	281/4,805 (5.85)	1,068/19,220 (5.56)	1.07 (0.93-1.22)	0.364	0.96 (0.86-1.08)	0.501
≥ 545 days	324/4,805 (6.74)	1,283/19,220 (6.68)	1.02 (0.90-1.16)	0.734	1.00 (0.89-1.11)	0.945
High income groups (n=25,575)						
Non-user	4,098/5,115 (80.12)	16,654/20,460 (81.40)	1		1	
< 180 days	312/5,115 (6.10)	1,132/20,460 (5.53)	1.12 (0.98-1.28)	0.087	0.86 (0.77-0.95)	0.005*
180–545 days	300/5,115 (5.87)	1,156/20,460 (5.65)	1.05 (0.92-1.20)	0.428	0.90 (0.81-1.00)	0.059
≥ 545 days	405/5,115 (7.92)	1,518/20,460 (7.42)	1.08 (0.97-1.22)	0.167	0.88 (0.80-0.97)	0.012*
Urban residents (n=22,235)						
Non-user	3,551/4,447 (79.85)	14,490/17,788 (81.46)	1		1	
< 180 days	279/4,447 (6.27)	977/17,788 (5.49)	1.17 (1.02-1.34)	0.03*	0.84 (0.75-0.94)	0.003*
180–545 days	278/4,447 (6.25)	1,020/17,788 (5.73)	1.11 (0.97-1.28)	0.13	0.88 (0.78-0.99)	0.029*
≥ 545 days	339/4,447 (7.62)	1,301/17,788 (7.31)	1.06 (0.94-1.20)	0.336	0.92 (0.83-1.03)	0.132
Rural residents (n=27,365)						
Non-user	4,449/5,473 (81.29)	17,959/21,892 (82.03)	1		1	
< 180 days	331/5,473 (6.05)	1,229/21,892 (5.61)	1.09 (0.96-1.23)	0.193	0.91 (0.82-1.02)	0.094
180–545 days	303/5,473 (5.54)	1,204/21,892 (5.5)	1.02 (0.89-1.16)	0.813	0.97 (0.87-1.08)	0.615
≥ 545 days	390/5,473 (7.13)	1,500/21,892 (6.85)	1.05 (0.93-1.18)	0.415	0.94 (0.85-1.04)	0.246
CCI scores = 0 (n=33,441)						
Non-user	5,284/6,331 (83.46)	22,958/27,110 (84.68)	1		1	
< 180 days	331/6,331 (5.23)	1,266/27,110 (4.67)	1.14 (1.00-1.29)	0.045*	0.86 (0.77-0.95)	0.003*
180–545 days	331/6,331 (5.23)	1,291/27,110 (4.76)	1.11 (0.98-1.26)	0.089	0.88 (0.80-0.98)	0.019*
≥ 545 days	385/6,331 (6.08)	1,595/27,110 (5.88)	1.05 (0.93-1.18)	0.418	0.96 (0.87-1.05)	0.386
CCI scores = 1 (n=10,037)						
Non-user	1,804/2,303 (78.33)	5,996/7,734 (77.53)	1		1	
< 180 days	159/2,303 (6.90)	526/7,734 (6.80)	1.00 (0.83-1.21)	0.96	0.91 (0.78-1.07)	0.256
180–545 days	139/2,303 (6.04)	541/7,734 (7.00)	0.85 (0.70-1.04)	0.11	1.05 (0.89-1.24)	0.541

≥ 545 days	201/2,303 (8.73)	671/7,734 (8.68)	1.00 (0.84-1.18)	0.959	0.88 (0.76-1.02)	0.085
CCI scores ≥ 2 (n=6,122)						
Non-user	912/1,286 (70.92)	3,495/4,836 (72.27)	1		1	
< 180 days	120/1,286 (9.33)	414/4,836 (8.56)	1.11 (0.90-1.38)	0.34	0.88 (0.74-1.06)	0.186
180–545 days	111/1,286 (8.63)	392/4,836 (8.11)	1.09 (0.87-1.36)	0.473	0.93 (0.77-1.12)	0.421
≥ 545 days	143/1,286 (11.12)	535/4,836 (11.06)	1.02 (0.84-1.25)	0.812	0.91 (0.77-1.09)	0.306
Non-diabetes history (n=32,845)						
Non-user	5,529/6,366 (86.85)	23,254/26,479 (87.82)	1		1	
< 180 days	313/6,366 (4.92)	1,139/26,479 (4.30)	1.16 (1.02-1.31)	0.027*	0.83 (0.75-0.93)	<0.001*
180–545 days	259/6,366 (4.07)	996/26,479 (3.76)	1.09 (0.95-1.26)	0.209	0.86 (0.77-0.97)	0.015*
≥ 545 days	265/6,366 (4.16)	1,090/26,479 (4.12)	1.02 (0.89-1.17)	0.751	0.92 (0.82-1.03)	0.169
Diabetes history (n=16,755)						
Non-user	2,471/3,554 (69.53)	9,195/13,201 (69.65)	1		1	
< 180 days	297/3,554 (8.36)	1,067/13,201 (8.08)	1.04 (0.90-1.19)	0.609	0.93 (0.83-1.05)	0.245
180–545 days	322/3,554 (9.06)	1,228/13,201 (9.30)	0.98 (0.86-1.11)	0.715	0.98 (0.88-1.10)	0.76
≥ 545 days	464/3,554 (13.06)	1,711/13,201 (12.96)	1.01 (0.90-1.13)	0.873	0.94 (0.86-1.04)	0.238
Non-dyslipidemia history (n=26,138)						
Non-user	5,045/5,321 (94.81)	19,937/20,817 (95.77)	1		1	
< 180 days	138/5,321 (2.59)	383/20,817 (1.84)	1.42 (1.17-1.73)	<0.001*	0.74 (0.63-0.88)	<0.001*
180–545 days	67/5,321 (1.26)	216/20,817 (1.04)	1.23 (0.93-1.62)	0.148	0.88 (0.71-1.11)	0.289
≥ 545 days	71/5,321 (1.33)	281/20,817 (1.35)	1.00 (0.77-1.30)	0.991	1.09 (0.88-1.34)	0.448
Dyslipidemia history (n=23,462)						
Non-user	2,955/4,599 (64.25)	12,512/18,863 (66.33)	1		1	
< 180 days	472/4,599 (10.26)	1,823/18,863 (9.66)	1.10 (0.98-1.22)	0.098	0.92 (0.84-1.01)	0.065
180–545 days	514/4,599 (11.18)	2,008/18,863 (10.65)	1.08 (0.98-1.20)	0.132	0.93 (0.85-1.01)	0.09
≥ 545 days	658/4,599 (14.31)	2,520/18,863 (13.36)	1.11 (1.01-1.22)	0.038*	0.91 (0.84-0.98)	0.014*

Abbreviations: CRC, colorectal cancer; OR, odds ratio; 95% CI, 95% confidence interval. * Significance at $p < 0.05$. †Adjusted for age, sex, income, region of residence, diabetes history, CCI, diabetes history, and dyslipidemia history.

Supplementary table 4. Crude and overlap propensity score weighted odd ratios of dates of hydrophilic statin prescription for colorectal cancer

Characteristics	N of Colorectal cancer (exposure/total, %)	N of Control (exposure/total, %)	ORs for CRC (95% CI)			
			Crude	p	Overlap weighted model †	p
Age < 65 years old (n=24,265)						
Non-user	4,611/4,853 (95.01)	18,493/19,412 (95.27)	1		1	
< 180 days	91/4,853 (1.88)	324/19,412 (1.67)	1.13 (0.89-1.42)	0.32	0.91 (0.75-1.10)	0.336
180–545 days	85/4,853 (1.75)	331/19,412 (1.71)	1.03 (0.81-1.31)	0.81	0.99 (0.82-1.21)	0.949
≥ 545 days	66/4,853 (1.36)	264/19,412 (1.36)	1.00 (0.76-1.32)	0.985	1.03 (0.83-1.28)	0.794
Age ≥ 65 years old (n=25,335)						
Non-user	4,599/5,067 (90.76)	18,457/20,268 (91.06)	1		1	
< 180 days	155/5,067 (3.06)	533/20,268 (2.63)	1.17 (0.97-1.40)	0.096	0.83 (0.72-0.97)	0.016*
180–545 days	133/5,067 (2.62)	583/20,268 (2.88)	0.92 (0.76-1.11)	0.365	1.06 (0.91-1.24)	0.434
≥ 545 days	180/5,067 (3.55)	695/20,268 (3.43)	1.04 (0.88-1.23)	0.65	0.93 (0.81-1.07)	0.31
Male (n=29,665)						
Non-user	5,544/5,933 (93.44)	22,175/23,732 (93.44)	1		1	
< 180 days	138/5,933 (2.33)	460/23,732 (1.94)	1.20 (0.99-1.45)	0.063	0.83 (0.71-0.98)	0.025*
180–545 days	127/5,933 (2.14)	505/23,732 (2.13)	1.01 (0.83-1.22)	0.953	0.99 (0.85-1.17)	0.937
≥ 545 days	124/5,933 (2.09)	592/23,732 (2.49)	0.84 (0.69-1.02)	0.077	1.20 (1.02-1.40)	0.023*
Female (n=19,935)						
Non-user	3,666/3,987 (91.95)	14,775/15,948 (92.64)	1		1	
< 180 days	108/3,987 (2.71)	397/15,948 (2.49)	1.10 (0.88-1.36)	0.403	0.89 (0.75-1.07)	0.212
180–545 days	91/3,987 (2.28)	409/15,948 (2.56)	0.90 (0.71-1.13)	0.353	1.10 (0.91-1.32)	0.325
≥ 545 days	122/3,987 (3.06)	367/15,948 (2.30)	1.34 (1.09-1.65)	0.006*	0.74 (0.62-0.88)	<0.001*
Low income groups (n=24,025)						
Non-user	4,462/4,805 (92.86)	17,950/19,220 (93.39)	1		1	
< 180 days	133/4,805 (2.77)	403/19,220 (2.10)	1.33 (1.09-1.62)	0.005*	0.76 (0.64-0.90)	0.001*
180–545 days	95/4,805 (1.98)	474/19,220 (2.47)	0.81 (0.65-1.01)	0.058	1.26 (1.06-1.50)	0.01*
≥ 545 days	115/4,805 (2.39)	393/19,220 (2.04)	1.18 (0.95-1.45)	0.129	0.86 (0.72-1.03)	0.097
High income groups (n=25,575)						
Non-user	4,748/5,115 (92.83)	19,000/20,460 (92.86)	1		1	
< 180 days	113/5,115 (2.21)	454/20,460 (2.22)	1.00 (0.81-1.23)	0.97	0.98 (0.82-1.15)	0.773
180–545 days	123/5,115 (2.40)	440/20,460 (2.15)	1.12 (0.91-1.37)	0.278	0.87 (0.74-1.03)	0.103
≥ 545 days	131/5,115 (2.56)	566/20,460 (2.77)	0.93 (0.76-1.12)	0.435	1.06 (0.91-1.24)	0.471
Urban residents (n=22,235)						
Non-user	4,121/4,447 (92.67)	16,567/17,788 (93.14)	1		1	
< 180 days	117/4,447 (2.63)	390/17,788 (2.19)	1.21 (0.98-1.49)	0.079	0.83 (0.69-0.98)	0.031*
180–545 days	106/4,447 (2.38)	394/17,788 (2.21)	1.08 (0.87-1.34)	0.479	0.91 (0.76-1.09)	0.319
≥ 545 days	103/4,447 (2.32)	437/17,788 (2.46)	0.95 (0.76-1.18)	0.627	1.05 (0.88-1.25)	0.62
Rural residents (n=27,365)						
Non-user	5,089/5,473 (92.98)	20,383/21,892 (93.11)	1		1	
< 180 days	129/5,473 (2.36)	467/21,892 (2.13)	1.11 (0.91-1.35)	0.315	0.89 (0.76-1.05)	0.158
180–545 days	112/5,473 (2.05)	520/21,892 (2.38)	0.86 (0.70-1.06)	0.161	1.15 (0.98-1.36)	0.088
≥ 545 days	143/5,473 (2.61)	522/21,892 (2.38)	1.10 (0.91-1.32)	0.332	0.91 (0.78-1.06)	0.24
CCI scores = 0 (n=33,441)						
Non-user	5,930/6,331 (93.67)	25,517/27,110 (94.12)	1		1	
< 180 days	141/6,331 (2.23)	496/27,110 (1.83)	1.22 (1.01-1.48)	0.037*	0.81 (0.69-0.94)	0.007*

180–545 days	136/6,331 (2.15)	541/27,110 (2.00)	1.08 (0.89-1.31)	0.418	0.93 (0.79-1.08)	0.322
≥ 545 days	124/6,331 (1.96)	556/27,110 (2.05)	0.96 (0.79-1.17)	0.682	1.06 (0.91-1.24)	0.474
CCI scores = 1 (n=10,037)						
Non-user	2,122/2,303 (92.14)	7,049/7,734 (91.14)	1		1	
< 180 days	65/2,303 (2.82)	208/7,734 (2.69)	1.04 (0.78-1.38)	0.796	0.89 (0.70-1.14)	0.366
180–545 days	49/2,303 (2.13)	231/7,734 (2.99)	0.70 (0.52-0.96)	0.028*	1.31 (1.01-1.69)	0.039*
≥ 545 days	67/2,303 (2.91)	246/7,734 (3.18)	0.90 (0.69-1.19)	0.475	0.99 (0.78-1.24)	0.899
CCI scores ≥ 2 (n=6,122)						
Non-user	1,158/1,286 (90.05)	4,384/4,836 (90.65)	1		1	
< 180 days	40/1,286 (3.11)	153/4,836 (3.16)	0.99 (0.69-1.41)	0.955	1.00 (0.75-1.34)	0.978
180–545 days	33/1,286 (2.57)	142/4,836 (2.94)	0.88 (0.60-1.29)	0.514	1.11 (0.81-1.51)	0.521
≥ 545 days	55/1,286 (4.28)	157/4,836 (3.25)	1.33 (0.97-1.82)	0.078	0.72 (0.55-0.95)	0.019*
Non-diabetes history (n=32,845)						
Non-user	6,076/6,366 (95.44)	25,215/26,479 (95.23)	1		1	
< 180 days	123/6,366 (1.93)	446/26,479 (1.68)	1.14 (0.94-1.40)	0.189	0.85 (0.72-1.00)	0.055
180–545 days	82/6,366 (1.29)	424/26,479 (1.60)	0.80 (0.63-1.02)	0.07	1.21 (1.00-1.45)	0.048*
≥ 545 days	85/6,366 (1.34)	394/26,479 (1.49)	0.90 (0.71-1.13)	0.358	1.08 (0.90-1.31)	0.398
Diabetes history (n=16,755)						
Non-user	3,134/3,554 (88.18)	11,735/13,201 (88.89)	1		1	
< 180 days	123/3,554 (3.46)	411/13,201 (3.11)	1.12 (0.91-1.38)	0.277	0.87 (0.73-1.03)	0.107
180–545 days	136/3,554 (3.83)	490/13,201 (3.71)	1.04 (0.86-1.26)	0.697	0.93 (0.79-1.10)	0.394
≥ 545 days	161/3,554 (4.53)	565/13,201 (4.28)	1.07 (0.89-1.28)	0.479	0.90 (0.77-1.05)	0.166
Non-dyslipidemia history (n=26,138)						
Non-user	5,213/5,321 (97.97)	20,491/20,817 (98.43)	1		1	
< 180 days	49/5,321 (0.92)	117/20,817 (0.56)	1.65 (1.18-2.30)	0.003*	0.64 (0.48-0.85)	0.002*
180–545 days	28/5,321 (0.53)	111/20,817 (0.53)	0.99 (0.65-1.50)	0.968	1.07 (0.77-1.50)	0.671
≥ 545 days	31/5,321 (0.58)	98/20,817 (0.47)	1.24 (0.83-1.86)	0.292	0.87 (0.62-1.22)	0.413
Dyslipidemia history (n=23,462)						
Non-user	3,997/4,599 (86.91)	16,459/18,863 (87.26)	1		1	
< 180 days	197/4,599 (4.28)	740/18,863 (3.92)	1.10 (0.93-1.29)	0.263	0.91 (0.80-1.04)	0.163
180–545 days	190/4,599 (4.13)	803/18,863 (4.26)	0.97 (0.83-1.15)	0.753	1.03 (0.90-1.17)	0.675
≥ 545 days	215/4,599 (4.67)	861/18,863 (4.56)	1.03 (0.88-1.20)	0.722	0.97 (0.86-1.10)	0.675

Abbreviations: CRC, colorectal cancer; OR, odds ratio; 95% CI, 95% confidence interval. * Significance at $p < 0.05$. †Adjusted for age, sex, income, region of residence, diabetes history, CCI, diabetes history, and dyslipidemia history.

Supplementary table 5. Crude and overlap propensity score weighted odd ratios of dates of any statin prescription for mortality in colorectal cancer

Characteristics	Dead patients	Survived patients	ORs for mortality (95% CI)			
	(exposure/total, %)	(exposure/total, %)	Crude	p	Overlap weighted model †	p
Age < 65 years old (n= 4,853)						
Non-user	929/1,103 (84.22)	3,058/3,750 (81.55)	1		1	
< 180 days	70/1,103 (6.35)	236/3,750 (6.29)	0.98 (0.74-1.29)	0.865	0.73 (0.57-0.93)	0.012*
180–545 days	60/1,103 (5.44)	209/3,750 (5.57)	0.94 (0.70-1.27)	0.708	0.76 (0.58-1.00)	0.048*
≥ 545 days	44/1,103 (3.99)	247/3,750 (6.59)	0.59 (0.42-0.81)	0.001*	1.24 (0.94-1.64)	0.13
Age ≥ 65 years old (n= 5,067)						
Non-user	1,837/2,470 (74.37)	1,697/2,597 (65.34)	1		1	
< 180 days	190/2,470 (7.69)	190/2,597 (7.32)	0.92 (0.75-1.14)	0.463	0.80 (0.64-1.00)	0.051
180–545 days	155/2,470 (6.28)	244/2,597 (9.40)	0.59 (0.47-0.73)	<0.001*	1.08 (0.87-1.35)	0.485
≥ 545 days	288/2,470 (11.66)	466/2,597 (17.94)	0.57 (0.49-0.67)	<0.001*	1.21 (1.02-1.45)	0.032*
Male (n= 5,933)						
Non-user	1,716/2,166 (79.22)	2,912/3,767 (77.30)	1		1	
< 180 days	161/2, 166 (7.43)	246/3,767 (6.53)	1.11 (0.90-1.37)	0.322	0.77 (0.62-0.95)	0.016*
180–545 days	114/2,166 (5.26)	238/3,767 (6.32)	0.81 (0.65-1.02)	0.079	1.01 (0.81-1.27)	0.904
≥ 545 days	175/2,166 (8.08)	371/3,767 (9.85)	0.80 (0.66-0.97)	0.021*	1.26 (1.03-1.53)	0.023*
Female (n= 3,987)						
Non-user	1,050/1,407 (74,63)	1,843/2,580 (71.43)	1		1	
< 180 days	99/1,407 (7.04)	180/2,580 (6,98)	0.97 (0.75-1.25)	0.788	0.79 (0.61-1.02)	0.075
180–545 days	101/1,407 (7.18)	215/2,580 (8.33)	0.82 (0.64-1.06)	0.128	0.87 (0.67-1.12)	0.271
≥ 545 days	157/1,407 (11.16)	342/2,580 (13.26)	0.81 (0.66-0.99)	0.038*	1.15 (0.93-1.42)	0.208
Low income groups (n= 4,805)						
Non-user	1,405/1,786 (78.67)	2,267/3,019 (75.09)	1		1	
< 180 days	136/1,786 (7.61)	210/3,019 (6.96)	1.04 (0.83-1.31)	0.703	0.75 (0.60-0.95)	0.017*
180–545 days	102/1,786 (5.71)	212/3,019 (7.02)	0.78 (0.61-0.99)	0.043*	1.03 (0.80-1.32)	0.812
≥ 545 days	143/1,786 (8.01)	330/3,019 (10.93)	0.70 (0.57-0.86)	<0.001*	1.31 (1.06-1.63)	0.013*
High income groups (n= 5,115)						
Non-user	1,361/1,787 (76.16)	2,488/3,328 (74.76)	1		1	
< 180 days	124/1,787 (6.94)	216/3,328 (6.49)	1.05 (0.83-1.32)	0.682	0.82 (0.65-1.03)	0.086
180–545 days	113/1,787 (6.32)	241/3,328 (7.24)	0.86 (0.68-1.08)	0.195	0.88 (0.70-1.11)	0.295
≥ 545 days	189/1,787 (10.58)	383/3,328 (11.51)	0.90 (0.75-1.09)	0.279	1.15 (0.94-1.40)	0.17
Urban residents (n= 4,447)						
Non-user	1,163/1,520 (76.51)	2,168/2,927 (74.07)	1		1	
< 180 days	108/1,520 (7.11)	208/2,927 (7.11)	0.97 (0.76-1.23)	0.793	0.90 (0.70-1.14)	0.377
180–545 days	96/1,520 (6.32)	227/2,927 (7.76)	0.79 (0.61-1.01)	0.061	1.00 (0.78-1.29)	0.981
≥ 545 days	153/1,520 (10.07)	324/2,927 (11.07)	0.88 (0.72-1.08)	0.223	1.12 (0.90-1.39)	0.294
Rural residents (n= 5,473)						
Non-user	1,603/2,053 (78.08)	2,587/3,420 (75.64)	1		1	
< 180 days	152/2,053 (7.40)	218/3,420 (6.37)	1.13 (0.91-1.40)	0.285	0.68 (0.54-0.85)	<0.001*
180–545 days	119/2,053 (5.80)	226/3,420 (6.61)	0.85 (0.67-1.07)	0.166	0.91 (0.72-1.15)	0.43
≥ 545 days	179/2,053 (8.72)	389/3,420 (11.37)	0.74 (0.62-0.90)	0.002*	1.30 (1.07-1.58)	0.008*
CCI scores = 0 (n= 6,331)						
Non-user	1,539/1,874 (82.12)	3,471/4,457 (77.88)	1		1	
< 180 days	106/1,874 (5.66)	278/4,457 (6.24)	0.86 (0.68-1.08)	0.202	0.79 (0.63-0.98)	0.035*

180–545 days	97/1,874 (5.18)	287/4,457 (6.44)	0.76 (0.60-0.97)	0.025*	0.86 (0.69-1.08)	0.205
≥ 545 days	132/1,874 (7.04)	421/4,457 (9.45)	0.71 (0.58-0.87)	<0.001*	1.05 (0.86-1.29)	0.617
CCI scores = 1 (n= 2,303)						
Non-user	752/976 (77.05)	924/1,327 (69.63)	1		1	
< 180 days	84/976 (6.61)	100/1,327 (7.54)	1.03 (0.76-1.40)	0.839	0.68 (0.49-0.95)	0.023*
180–545 days	51/976 (5.23)	109/1,327 (8.21)	0.57 (0.41-0.81)	0.002*	1.14 (0.80-1.61)	0.463
≥ 545 days	89/976 (9.12)	194/1,327 (14.62)	0.56 (0.43-0.74)	<0.001*	1.47 (1.11-1.96)	0.007*
CCI scores ≥ 2 (n= 1,286)						
Non-user	475/723 (65.70)	360/563 (63.94)	1		1	
< 180 days	70/723 (9.68)	48/563 (8.53)	1.11 (0.75-1.64)	0.617	0.86 (0.58-1.28)	0.462
180–545 days	67/723 (9.27)	57/563 (10.12)	0.89 (0.61-1.30)	0.55	0.94 (0.64-1.38)	0.754
≥ 545 days	111/723 (15.35)	98/563 (17.41)	0.86 (0.63-1.16)	0.325	1.21 (0.87-1.68)	0.257
Non-diabetes history (n= 6,366)						
Non-user	1,905/2,246 (84.82)	3,418/4,120 (82.96)	1		1	
< 180 days	134/2,246 (5.97)	236/4,120 (5.73)	1.02 (0.82-1.27)	0.868	0.77 (0.62-0.97)	0.025*
180–545 days	97/2,246 (4.32)	202/4,120 (4.90)	0.86 (0.67-1.10)	0.24	0.90 (0.70-1.15)	0.393
≥ 545 days	110/2,246 (4.90)	264/4,120 (6.41)	0.75 (0.59-0.94)	0.013*	1.25 (0.99-1.59)	0.059
Diabetes history (n= 3,554)						
Non-user	861/1,327 (64.88)	1,337/2,227 (60.04)	1		1	
< 180 days	126/1,327 (9.50)	190/2,227 (8.53)	1.03 (0.81-1.31)	0.811	0.80 (0.63-1.02)	0.07
180–545 days	118/1,327 (8.89)	251/2,227 (11.27)	0.73 (0.58-0.92)	0.009*	1.01 (0.80-1.28)	0.901
≥ 545 days	222/1,327 (16.73)	449/2,227 (20.16)	0.77 (0.64-0.92)	0.004*	1.19 (0.99-1.44)	0.064
Non-dyslipidemia history (n= 5,321)						
Non-user	2,177/2,371 (91.82)	2,788/2,950 (94.51)	1		1	
< 180 days	104/2,371 (4.39)	61/2,950 (2.07)	2.18 (1.58-3.01)	<0.001*	0.72 (0.53-0.99)	0.043*
180–545 days	40/2,371 (1.69)	43/2,950 (1.46)	1.19 (0.77-1.84)	0.429	1.46 (0.94-2.27)	0.092
≥ 545 days	50/2,371 (2.11)	58/2,950 (1.97)	1.10 (0.75-1.62)	0.612	2.02 (1.35-3.02)	<0.001*
Dyslipidemia history (n= 4,599)						
Non-user	589/1,202 (49.00)	1,967/3,397 (57.90)	1		1	
< 180 days	156/1,202 (12.98)	365/3,397 (10.74)	1.43 (1.16-1.76)	<0.001*	0.77 (0.64-0.93)	0.007*
180–545 days	175/1,202 (14.56)	410/3,397 (12.07)	1.43 (1.17-1.74)	<0.001*	0.86 (0.72-1.02)	0.088
≥ 545 days	282/1,202 (23.46)	655/3,397 (19.28)	1.44 (1.22-1.70)	<0.001*	1.09 (0.93-1.26)	0.282

Abbreviations: OR, odds ratio; 95% CI, 95% confidence interval. * Significance at p <0.05. †Adjusted for age, sex, income, region of residence, diabetes history, CCI, diabetes history, and dyslipidemia history.

Supplementary table 6. Crude and overlap propensity score weighted odd ratios of dates of lipophilic statin prescription for mortality in colorectal cancer

Characteristics	Dead patients	Survived patients	ORs for mortality (95% CI)			
	(exposure/total, %)	(exposure/total, %)	Crude	p	Overlap weighted model †	p
Age < 65 years old (n= 4,853)						
Non-user	947/1,103 (85.86)	3,202/3,750 (85.39)	1		1	
< 180 days	67/1,103 (6.07)	206/3,750 (5.49)	1.10 (0.83-1.46)	0.513	0.62 (0.48-0.80)	<0.001*
180–545 days	56/1,103 (5.08)	176/3,750 (4.69)	1.08 (0.79-1.47)	0.643	0.66 (0.49-0.87)	0.003*
≥ 545 days	33/1,103 (2.99)	166/3,750 (4.43)	0.67 (0.46-0.98)	0.041*	1.03 (0.74-1.43)	0.858
Age ≥ 65 years old (n= 5,067)						
Non-user	1,939/2,470 (78.50)	1,912/2,597 (73.62)	1		1	
< 180 days	173/2,470 (7.00)	164/2,597 (6.31)	1.04 (0.83-1.30)	0.729	0.73 (0.58-0.92)	0.008*
180–545 days	145/2,470 (5.87)	204/2,597 (7.86)	0.70 (0.56-0.88)	0.002*	0.88 (0.70-1.10)	0.26
≥ 545 days	213/2,470 (8.62)	317/2,597 (12.21)	0.66 (0.55-0.80)	<0.001*	0.99 (0.82-1.21)	0.954
Male (n= 5,933)						
Non-user	1,790/2,166 (82.64)	3,101/3,767 (82.32)	1		1	
< 180 days	142/2,166 (6.56)	216/3,767 (5.73)	1.14 (0.91-1.42)	0.246	0.76 (0.60-0.95)	0.015*
180–545 days	104/2,166 (4.80)	200/3,767 (5.31)	0.90 (0.71-1.15)	0.402	0.89 (0.70-1.13)	0.345
≥ 545 days	130/2,166 (6.00)	250/3,767 (6.64)	0.90 (0.72-1.12)	0.352	1.09 (0.88-1.37)	0.428
Female (n= 3,987)						
Non-user	1,096/1,407 (77.90)	2,013/2,580 (78.02)	1		1	
< 180 days	98/1,407 (6.97)	154/2,580 (5.97)	1.17 (0.90-1.52)	0.246	0.60 (0.46-0.79)	<0.001*
180–545 days	97/1,407 (6.89)	180/2,580 (6.98)	0.99 (0.76-1.28)	0.938	0.67 (0.51-0.87)	0.002*
≥ 545 days	116/1,407 (8.24)	233/2,580 (9.03)	0.91 (0.72-1.16)	0.455	0.88 (0.70-1.12)	0.309
Low income groups (n= 4,805)						
Non-user	1,465/1,786 (82.03)	2,437/3,019 (80.72)	1		1	
< 180 days	122/1,786 (6.83)	176/3,019 (5.83)	1.15 (0.91-1.47)	0.244	0.68 (0.53-0.87)	0.002*
180–545 days	98/1,786 (5.49)	183/3,019 (6.06)	0.89 (0.69-1.15)	0.372	0.87 (0.67-1.13)	0.3
≥ 545 days	101/1,786 (5.66)	223/3,019 (7.39)	0.75 (0.59-0.96)	0.023*	1.16 (0.91-1.48)	0.227
High income groups (n= 5,115)						
Non-user	1,421/1,787 (79.52)	2,677/3,328 (80.44)	1		1	
< 180 days	118/1,787 (6.60)	194/3,328 (5.83)	1.15 (0.90-1.45)	0.262	0.71 (0.56-0.91)	0.006*
180–545 days	103/1,787 (5.76)	197/3,328 (5.92)	0.98 (0.77-1.26)	0.904	0.71 (0.56-0.91)	0.007*
≥ 545 days	145/1,787 (8.11)	260/3,328 (7.81)	1.05 (0.85-1.30)	0.65	0.89 (0.71-1.10)	0.284
Urban residents (n= 4,447)						
Non-user	1,215/1,520 (79.93)	2,336/2,927 (79.81)	1		1	
< 180 days	102/1,520 (6.71)	177/2,927 (6.05)	1.11 (0.86-1.43)	0.428	0.77 (0.60-0.99)	0.044*
180–545 days	87/1,520 (5.72)	191/2,927 (6.53)	0.88 (0.67-1.14)	0.322	0.85 (0.65-1.10)	0.224
≥ 545 days	116/1,520 (7.63)	223/2,927 (7.62)	1.00 (0.79-1.26)	0.999	0.93 (0.73-1.18)	0.552
Rural residents (n= 5,473)						
Non-user	1,671/2,053 (81.39)	2,778/3,420 (81.23)	1		1	
< 180 days	138/2,053 (6.72)	193/3,420 (5.64)	1.19 (0.95-1.49)	0.135	0.62 (0.49-0.79)	<0.001*
180–545 days	114/2,053 (5.55)	189/3,420 (5.53)	1.00 (0.79-1.28)	0.982	0.74 (0.58-0.94)	0.015*
≥ 545 days	130/2,053 (6.33)	260/3,420 (7.60)	0.83 (0.67-1.03)	0.098	1.07 (0.86-1.33)	0.569
CCI scores = 0 (n= 6,331)						
Non-user	1,586/1,874 (84.63)	3,698/4,457 (82.97)	1		1	
< 180 days	93/1,874 (4.96)	238/4,457 (5.34)	0.91 (0.71-1.17)	0.46	0.65 (0.51-0.83)	<0.001*
180–545 days	93/1,874 (4.96)	238/4,457 (5.34)	0.91 (0.71-1.17)	0.46	0.70 (0.55-0.89)	0.003*
≥ 545 days	102/1,874 (5.44)	283/4,457 (6.35)	0.84 (0.67-1.06)	0.145	0.81 (0.65-1.02)	0.076
CCI scores = 1 (n= 2,303)						
Non-user	790/976 (80.94)	1,014/1,327 (76.41)	1		1	
< 180 days	77/976 (7.89)	82/1,327 (6.18)	1.21 (0.87-1.67)	0.26	0.60 (0.42-0.85)	0.004*
180–545 days	46/976 (4.71)	93/1,327 (7.01)	0.63 (0.44-0.91)	0.015*	1.00 (0.69-1.44)	1

≥ 545 days	63/976 (6.45)	138/1,327 (10.4)	0.59 (0.43-0.80)	<0.001*	1.39 (1.02-1.90)	0.037*
CCI scores ≥ 2 (n= 1,286)						
Non-user	510/723 (70.54)	402/563 (71.4)	1		1	
< 180 days	70/723 (9.68)	50/563 (8.88)	1.10 (0.75-1.62)	0.618	0.85 (0.58-1.25)	0.411
180–545 days	62/723 (8.58)	49/563 (8.70)	1.00 (0.67-1.48)	0.99	0.79 (0.53-1.17)	0.243
≥ 545 days	81/723 (11.2)	62/563 (11.01)	1.03 (0.72-1.47)	0.872	0.94 (0.65-1.36)	0.734
Non-diabetes history (n= 6,366)						
Non-user	1,956/2,246 (87.09)	3,573/4,120 (86.72)	1		1	
< 180 days	115/2,246 (5.12)	198/4,120 (4.81)	1.06 (0.84-1.34)	0.624	0.73 (0.57-0.92)	0.01*
180–545 days	92/2,246 (4.10)	167/4,120 (4.05)	1.01 (0.78-1.31)	0.962	0.70 (0.54-0.92)	0.009*
≥ 545 days	83/2,246 (3.70)	182/4,120 (4.42)	0.83 (0.64-1.09)	0.177	1.03 (0.79-1.34)	0.825
Diabetes history (n= 3,554)						
Non-user	930/1,327 (70.08)	1,541/2,227 (69.20)	1		1	
< 180 days	125/1,327 (9.42)	172/2,227 (7.72)	1.20 (0.94-1.54)	0.136	0.67 (0.52-0.86)	0.002*
180–545 days	109/1,327 (8.21)	213/2,227 (9.56)	0.85 (0.66-1.08)	0.187	0.87 (0.69-1.11)	0.271
≥ 545 days	163/1,327 (12.28)	301/2,227 (13.52)	0.90 (0.73-1.10)	0.305	0.98 (0.79-1.20)	0.829
Non-dyslipidemia history (n= 5,321)						
Non-user	2,209/2,371 (93.17)	2,836/2,950 (96.14)	1		1	
< 180 days	92/2,371 (3.88)	46/2,950 (1.56)	2.57 (1.79-3.67)	<0.001*	0.65 (0.46-0.92)	0.016*
180–545 days	36/2,371 (1.52)	31/2,950 (1.05)	1.49 (0.92-2.42)	0.105	1.08 (0.66-1.76)	0.765
≥ 545 days	34/2,371 (1.43)	37/2,950 (1.25)	1.18 (0.74-1.89)	0.49	1.78 (1.11-2.84)	0.017*
Dyslipidemia history (n= 4,599)						
Non-user	677/1,202 (56.32)	2,278/3,397 (67.06)	1		1	
< 180 days	148/1,202 (12.31)	324/3,397 (9.54)	1.54 (1.24-1.90)	<0.001*	0.69 (0.57-0.84)	<0.001*
180–545 days	165/1,202 (13.73)	349/3,397 (10.27)	1.59 (1.30-1.95)	<0.001*	0.74 (0.62-0.89)	0.001*
≥ 545 days	212/1,202 (17.64)	446/3,397 (13.13)	1.60 (1.33-1.92)	<0.001*	0.90 (0.76-1.06)	0.217

Abbreviations: OR, odds ratio; 95% CI, 95% confidence interval. * Significance at p <0.05. †Adjusted for age, sex, income, region of residence, diabetes history, CCI, diabetes history, and dyslipidemia history.

Supplementary table 7. Crude and overlap propensity score weighted odd ratios of dates of hydrophilic statin prescription for mortality in colorectal cancer

Characteristics	Dead patients	Survived patients	ORs for mortality (95% CI)			
	(exposure/total, %)	(exposure/total, %)	Crude	p	Overlap weighted model †	p
Age < 65 years old (n= 4,853)						
Non-user	1,069/1,103 (96.92)	3,542/3,750 (94.45)	1		1	
< 180 days	16/1,103 (1.45)	75/3,750 (2.00)	0.71 (0.41-1.22)	0.211	1.25 (0.80-1.97)	0.330
180–545 days	11/1,103 (1.00)	74/3,750 (1.97)	0.49 (0.26-0.93)	0.029*	1.70 (1.04-2.77)	0.034*
≥ 545 days	7/1,103 (0.63)	59/3,750 (1.57)	0.39 (0.18-0.86)	0.02*	2.01 (1.11-3.63)	0.021*
Age ≥ 65 years old (n= 5,067)						
Non-user	2,315/2,470 (93.72)	2,284/2,597 (87.95)	1		1	
< 180 days	56/2,470 (2.27)	99/2,597 (3.81)	0.56 (0.40-0.78)	<0.001*	1.46 (1.04-2.03)	0.028*
180–545 days	38/2,470 (1.54)	95/2,597 (3.66)	0.39 (0.27-0.58)	<0.001*	1.77 (1.21-2.60)	0.003*
≥ 545 days	61/2,470 (2.47)	119/2,597 (4.58)	0.51 (0.37-0.69)	<0.001*	1.75 (1.27-2.41)	<0.001*
Male (n= 5,933)						
Non-user	2,056/2,166 (94.92)	3,488/3,767 (92.59)	1		1	
< 180 days	44/2,166 (2.03)	94/3,767 (2.50)	0.79 (0.55-1.14)	0.212	1.28 (0.91-1.80)	0.162
180–545 days	33/2, 166 (1.52)	94/3,767 (2.50)	0.60 (0.40-0.89)	0.011*	1.38 (0.95-1.99)	0.090
≥ 545 days	33/2,166 (1.52)	91/3,767 (2.42)	0.62 (0.41-0.92)	0.018*	1.80 (1.23-2.65)	0.003*
Female (n= 3,987)						
Non-user	1,328/1,407 (94.39)	2,338/2,580 (90.62)	1		1	
< 180 days	28/1,407 (1.99)	80/2,580 (3.1)	0.62 (0.40-0.95)	0.029*	1.62 (1.06-2.46)	0.024*
180–545 days	16/1,407 (1.14)	75/2,580 (2.91)	0.38 (0.22-0.65)	<0.001*	2.67 (1.58-4.52)	<0.001*
≥ 545 days	35/1,407 (2.49)	87/2,580 (3.37)	0.71 (0.48-1.05)	0.089	1.81 (1.23-2.65)	0.003*
Low income groups (n= 4,805)						
Non-user	1,695/1,786 (94.9)	2,767/3,019 (91.65)	1		1	
< 180 days	38/1,786 (2.13)	95/3,019 (3.15)	0.65 (0.45-0.96)	0.028*	1.50 (1.04-2.16)	0.029*
180–545 days	18/1,786 (1.01)	77/3,019(2.55)	0.38 (0.23-0.64)	<0.001*	2.07 (1.29-3.33)	0.002*
≥ 545 days	35/1,786 (1.96)	80/3,019 (2.65)	0.71 (0.48-1.07)	0.101	1.47 (0.99-2.20)	0.057
High income groups (n= 5,115)						
Non-user	1,689/1,787 (94.52)	3,059/3,328 (91.92)	1		1	
< 180 days	34/1,787 (1.90)	79/3,328 (2.37)	0.78 (0.52-1.17)	0.23	1.30 (0.88-1.91)	0.187
180–545 days	31/1,787 (1.73)	92/3,328 (2.76)	0.61 (0.40-0.92)	0.019*	1.53 (1.04-2.25)	0.031*
≥ 545 days	33/1,787 (1.85)	98/3,328 (2.94)	0.61 (0.41-0.91)	0.015*	2.21 (1.52-3.21)	<0.001*
Urban residents (n= 4,447)						
Non-user	1,435/1,520 (94.41)	2,686/2,927 (91.77)	1		1	
< 180 days	31/1,520 (2.04)	86/2,927 (2.94)	0.67 (0.45-1.02)	0.064	1.51 (1.02-2.23)	0.041*
180–545 days	28/1,520 (1.84)	78/2,927 (2.66)	0.67 (0.43-1.04)	0.074	1.28 (0.85-1.94)	0.232
≥ 545 days	26/1,520 (1.71)	77/2,927 (2.63)	0.63 (0.40-0.99)	0.045*	1.84 (1.21-2.79)	0.004*
Rural residents (n= 5,473)						
Non-user	1,949/2,053 (94.93)	3,140/3,420 (91.81)	1		1	
< 180 days	41/2,053 (2.00)	88/3,420 (2.57)	0.75 (0.52-1.09)	0.134	1.32 (0.92-1.89)	0.13
180–545 days	21/2,053 (1.02)	91/3,420 (2.66)	0.37 (0.23-0.60)	<0.001*	2.36 (1.52-3.67)	<0.001*
≥ 545 days	42/2,053 (2.05)	101/3,420 (2.95)	0.67 (0.47-0.96)	0.031*	1.82 (1.27-2.61)	0.001*
CCI scores = 0 (n= 6,331)						
Non-user	1,799/1,874 (96.00)	4,131/4,457 (92.69)	1		1	
< 180 days	32/1,874 (1.71)	109/4,457 (2.45)	0.67 (0.45-1.00)	0.052	1.46 (1.02-2.09)	0.04*
180–545 days	21/1,874 (1.12)	115/4,457 (2.58)	0.42 (0.26-0.67)	<0.001*	1.66 (1.13-2.45)	0.01*

≥ 545 days	22/1,874 (1.17)	102/4,457 (2.29)	0.50 (0.31-0.79)	0.003*	1.91 (1.29-2.85)	0.001*
CCI scores = 1 (n= 2,303)						
Non-user	924/976 (94.67)	1,198/1,327 (90.28)	1		1	
< 180 days	17/976 (1.74)	48/1,327 (3.62)	0.46 (0.26-0.80)	0.006*	1.59 (0.93-2.73)	0.089
180–545 days	12/976 (1.23)	37/1,327 (2.79)	0.42 (0.22-0.81)	0.01*	1.81 (0.95-3.45)	0.07
≥ 545 days	23/976 (2.36)	44/1,327 (3.32)	0.68 (0.41-1.13)	0.136	1.40 (0.83-2.35)	0.212
CCI scores ≥ 2 (n= 1,286)						
Non-user	661/723 (91.42)	497/563 (88.28)	1		1	
< 180 days	23/723 (3.18)	17/563 (3.02)	1.02 (0.54-1.92)	0.958	1.07 (0.59-1.95)	0.823
180–545 days	16/723 (2.21)	17/563 (3.02)	0.71 (0.35-1.41)	0.328	1.59 (0.79-3.22)	0.196
≥ 545 days	23/723 (3.18)	32/563 (5.68)	0.54 (0.31-0.94)	0.028*	2.17 (1.22-3.86)	0.009*
Non-diabetes history (n= 6,366)						
Non-user	2,166/2,246 (96.44)	3,910/4,120 (94.9)	1		1	
< 180 days	41/2,246 (1.83)	82/4,120 (1.99)	0.90 (0.62-1.32)	0.596	1.16 (0.80-1.68)	0.448
180–545 days	19/2,246 (0.85)	63/4,120 (1.53)	0.54 (0.33-0.91)	0.021*	1.76 (1.09-2.86)	0.022*
≥ 545 days	20/2,246 (0.89)	65/4,120 (1.58)	0.56 (0.34-0.92)	0.022*	2.21 (1.35-3.59)	0.001*
Diabetes history (n= 3,554)						
Non-user	1,218/1,327 (91.79)	1,916/2,227 (86.04)	1		1	
< 180 days	31/1,327 (2.34)	92/2,227 (4.13)	0.53 (0.35-0.80)	0.003*	1.72 (1.17-2.51)	0.005*
180–545 days	30/1,327 (2.26)	106/2,227 (4.76)	0.45 (0.29-0.67)	<0.001*	1.72 (1.17-2.51)	0.005*
≥ 545 days	48/1,327 (3.62)	113/2,227 (5.07)	0.67 (0.47-0.94)	0.022*	1.66 (1.19-2.32)	0.003*
Non-dyslipidemia history (n= 5,321)						
Non-user	2,322/2,371 (97.93)	2,891/2,950 (98.00)	1		1	
< 180 days	26/2,371 (1.10)	23/2,950 (0.78)	1.41 (0.80-2.47)	0.235	1.09 (0.63-1.89)	0.749
180–545 days	9/2,371 (0.38)	19/2,950 (0.64)	0.59 (0.27-1.31)	0.193	2.60 (1.14-5.88)	0.022*
≥ 545 days	14/2,371 (0.59)	17/2,950 (0.58)	1.03 (0.50-2.08)	0.945	2.65 (1.17-6.00)	0.019*
Dyslipidemia history (n= 4,599)						
Non-user	1,062/1,202 (88.35)	2,935/3,397 (86.4)	1		1	
< 180 days	46/1,202 (3.83)	151/3,397 (4.45)	0.84 (0.60-1.18)	0.318	1.50 (1.12-2.01)	0.006*
180–545 days	40/1,202 (3.33)	150/3,397 (4.42)	0.74 (0.52-1.05)	0.093	1.60 (1.18-2.17)	0.002*
≥ 545 days	54/1,202 (4.49)	161/3,397 (4.74)	0.93 (0.68-1.27)	0.638	1.70 (1.29-2.23)	<0.001*

Abbreviations: OR, odds ratio; 95% CI, 95% confidence interval. * Significance at $p < 0.05$. †Adjusted for age, sex, income, region of residence, diabetes history, CCI, diabetes history, and dyslipidemia history.
